# Unmet needs in uncomplicated urinary tract infection in the United States and Germany: a physician survey

**DOI:** 10.1186/s12879-023-08207-x

**Published:** 2023-05-03

**Authors:** Megan O’Brien, Alen Marijam, Fanny S. Mitrani-Gold, Laura Terry, Gavin Taylor-Stokes, Ashish V. Joshi

**Affiliations:** 1 Adelphi Real World, Macclesfield, Cheshire UK; 2 GSK, Wavre, Belgium; 3 GSK, Collegeville, PA USA

**Keywords:** Antibiotics, Uncomplicated urinary tract infection, Primary care, Secondary care, Survey

## Abstract

**Background:**

Uncomplicated urinary tract infections (uUTIs/acute cystitis) are among the most common infections in women worldwide. There are differences in uUTI treatment guidelines between countries and understanding the needs of physicians in diverse healthcare systems is important for developing new treatments. We performed a survey of physicians in the United States (US) and Germany to understand their perceptions of, and management approaches to uUTI.

**Methods:**

This was a cross-sectional online survey of physicians in the US and Germany who were actively treating patients with uUTI (≥ 10 patients/month). Physicians were recruited via a specialist panel and the survey was piloted with 2 physicians (1 US, 1 Germany) prior to study commencement. Data were analyzed with descriptive statistics.

**Results:**

A total of 300 physicians were surveyed (*n* = 200 US, *n* = 100 Germany). Across countries and specialties, physicians estimated 16–43% of patients did not receive complete relief from initial therapy and 33–37% had recurrent infections. Urine culture and susceptibility testing was more common in the US and among urologists. The most commonly selected first-line therapy was trimethoprim-sulfamethoxazole in the US (76%) and fosfomycin in Germany (61%). Ciprofloxacin was the most selected following multiple treatment failures (51% US, 45% Germany). Overall, 35% of US and 45% of German physicians agreed with the statement “I feel there is a good selection of treatment options” and ≥ 50% felt that current treatments provided good symptom relief. More than 90% of physicians included symptom relief amongst their top 3 treatment goals. The overall impact of symptoms on patients’ lives was rated “a great deal” by 51% of US and 38% of German physicians, increasing with each treatment failure. Most physicians (> 80%) agreed that antimicrobial resistance (AMR) is serious, but fewer (56% US, 46% Germany) had a high level of confidence in their knowledge of AMR.

**Conclusions:**

Treatment goals for uUTI were similar in the US and Germany, although with nuances to disease management approaches. Physicians recognized that treatment failures have a significant impact on patients’ lives and that AMR is a serious problem, though many did not have confidence in their own knowledge of AMR.

**Supplementary Information:**

The online version contains supplementary material available at 10.1186/s12879-023-08207-x.

## Background

Uncomplicated urinary tract infections (uUTI) are one of the most common bacterial infections in women and a key reason for antibiotic prescribing in the outpatient setting [1, 2]. Antimicrobial resistance (AMR) has emerged at the community level and has been highlighted by the World Health Organization as a major threat to health worldwide [3]. Treatment guidelines for uUTI exist at the national and international level, and, while broadly similar, there are differences in the recommendations and diagnostic and treatment practices based on geography [2, 4–6]. AMR is a global problem and the prevalence of uUTIs is generally considered to be high among women regardless of their location [1, 3, 7].

Because uUTIs are so common, many physicians in different care settings are involved in the management of patients. The methods by which these physicians diagnose and treat uUTI affect patient care, health care costs, and the likelihood of AMR [8]. It has also been reported that differences of opinion, or misunderstandings, between physicians and patients can lead to barriers to effective uUTI management [9, 10]. Such misunderstandings can often be explained by a lack of effective communication between the patient and the physician [10, 11]. Previous surveys have found that physicians vary in their approach to the management of uUTI [4] and that the specialty of the prescribing physician may influence prescribing patterns [12]. As such, understanding the perspectives of physicians from diverse healthcare systems is important for developing effective treatment strategies. An understanding of physicians’ management approaches and prescribing patterns should help increase the likelihood of the success of interventions developed to optimize the management of uUTI.

This study reports the results from a cross-sectional online survey of physicians (primary and secondary care) in the United States (US) and Germany, conducted to better understand their approaches to the treatment of uUTI.

## Methods

### Study design

We performed a cross-sectional, online survey of physicians in the US and Germany, recruited via a global physician panel (Leaders in Medicine Atlas [Medefield, United Kingdom]). To focus the cohort on physicians treating patients with uUTI, the following inclusion and exclusion criteria were used:

- Physicians residing in the US or Germany were included if they were actively managing patients with uUTI, had a minimum caseload of 10 patients with uUTI per month, and were responsible for making treatment decisions for these patients
- Specialties were a mix of: primary care physicians (PCP); urologists (URO), URO/gynecologists (GYN); GYN; obstetrician (OB)/GYN; and infectious disease (ID) specialists (US only)
- Physicians were excluded if they qualified/were board certified before 1980 or after 2016
- In the US, physicians were excluded if they were state government employees; Pharmacy & Therapeutic Committee Members; or licensed to practice medicine in or residents of Maine, Massachusetts, Minnesota, or Vermont.

After screening, physicians completed a 30-min online survey, which captured information on demographics and clinical characteristics, in addition to information relating to the study objectives. Physicians were compensated in line with fair-market value for the time needed to complete the survey.

Definitions of uUTI were provided to participating US and German physicians per guidelines as follows. In the US survey, uUTI was defined as acute cystitis occurring in patients with no functional or anatomical anomalies of the urinary tract, no renal function impairment, and no concomitant comorbidities associated with complicated urinary tract infection (UTI) [2, 13]. In the German survey, uUTI was defined as acute, sporadic, or recurrent lower- (uncomplicated cystitis) and/or upper- (uncomplicated pyelonephritis) UTI, limited to non-pregnant, premenopausal women with no known anatomical and functional abnormalities within the urinary tract or complicating comorbidities [6].

Physicians’ awareness of AMR was assessed by collating their responses to a series of statements (graded on a 7-point Likert scale, 1 = strongly disagree, 7 = strongly agree), and categorizing according to Björkman et al.: Group A – “no problem with antibiotic resistance, have never seen”; Group B – “the problem is bigger somewhere else”; Group C – “the development of AMR is serious and we must be careful” [14].

### Survey development and pilot

The survey was developed from 3 sources. These were a previous patient survey, review of published literature, and physician responses to a short survey (conducted by Medefield), which included an open-ended question on treatment goals in the management of uUTI. The survey was piloted with 1 physician from the US and 1 physician from Germany prior to recruitment. These physicians were recruited via the physician panel as described above and the pilot data were not included in the final results. The pilot served as a validation stage to ensure that the response options and the wording of the questions were appropriate and clear. Following the pilot, the study team made minor amendments to the survey, including tailoring to the approved antimicrobials available in each region. The final survey is included in the supplementary materials.

### Study objectives

The primary study objective was to understand physicians’ treatment goals, management approaches, and prescribing patterns for treating uUTI in the US and Germany. The secondary objectives of the study included understanding physicians’ perceptions of the impact of uUTI on patients, including symptoms, functional and emotional burden, quality of life, work and productivity, and financial impact. A further objective was to assess physician awareness of AMR and prescribing, and to evaluate the access physicians have to information on local, regional, and national antibiotic resistance data in the US and Germany.

### Statistical analysis

This was an exploratory survey, and no formal hypothesis was tested. Data were analyzed with descriptive statistics; for numeric data, the statistics presented include number of non-missing values, mean, standard deviation, range, median, and quartiles. Categorical data are presented as percentage of physicians in each category unless otherwise stated.

## Results

### Physician demographics and caseload

A total of 300 physicians were surveyed, comprising 200 from the US and 100 from Germany. Physician demographics are provided in Table 1; the majority of physicians from the US were from private practice (62%), whilst physicians from Germany were predominantly from a publicly funded, office-based practice (83%). Patients with uUTI formed approximately 11% of the total workload of physicians in the US and Germany (Table 1). The highest proportion of workload taken up by uUTI was for outpatient internists in the US (15%) and URO in Germany (21%). Physicians reported that the plurality of patients they treated for uUTI were aged 18–54 years (35% of uUTI caseload for both US and Germany physicians), followed by those aged over 65 years (32% of uUTI caseload for US and 31% for German physicians).

**Table 1:** Physician demographics and caseloads

	**US** **(*n* = 200)**	**Germany** **(*n* = 100)**
**Physician specialty**	**%**	**%**
General practice, primary care, emergency room	26	45
GYN, OB/GYN, URO/GYN	28	28
URO	17	22
Internist (outpatient)	11	3
Internist (inpatient)	15	2
Infectious disease^a^	4	–
**Physician care setting**	**%**	**%**
Office-based practice (public)	0	83
Private practice^a^	62	–
University/teaching hospital	10	12
Regional/community hospital	17	3
Health center	4	1
Regional center	2	2
Private hospital	3	0
Urgent care	2	0
**Patient caseload**^**b**^	**mean (%)**	**mean (%)**
**Total patient caseload**	**357.7**	**418.1**
General practice, primary care, emergency room	440.1	507.5
GYN, OB/GYN, URO/GYN	321.2	350.7
URO	339.0	331.7
Internist (outpatient)	310.2	300.0
Internist (inpatient)	364.1	416.7
Infectious disease^a^	258.8	–
**Total complicated UTI caseload**	**24.9 (7.0)**	**21.8 (5.2)**
General practice, primary care, emergency room	26.3 (6.0)	18.2 (3.6)
GYN, OB/GYN, URO/GYN	10.6 (3.3)	17.1 (4.9)
URO	41.4 (12.2)	36.5 (11.0)
Internist (outpatient)	33.6 (10.8)	16.5 (5.5)
Internist (inpatient)	15.5 (4.3)	16.7 (4.0)
Infectious disease^a^	55.4 (21.4)	–
**Total uncomplicated UTI caseload**	**40.9 (11.4)**	**47.0 (11.2)**
General practice, primary care, emergency room	48.3 (11.0)	49.7 (9.8)
GYN, OB/GYN, URO/GYN	32.0 (10.0)	29.9 (8.5)
URO	48.4 (14.3)	68.1 (20.5)
Internist (outpatient)	47.1 (15.2)	29.0 (9.7)
Internist (inpatient)	32.6 (9.0)	25.0 (6.0)
Infectious disease^a^	35.9 (13.9)	–
**Proportion of time spent in clinic seeing patients with uUTI**	**%**	**%**
General practice, primary care, emergency room	29	36
GYN, OB/GYN, URO/GYN	23	35
URO	36	51
Internist (outpatient)	35	23
Internist (inpatient)	30	15
Infectious disease^a^	28	–
**Age range of patients treated**	**%**	**%**
> 65 years	32	31
55–65 years	25	24
18–54 years	35	35
12–17 years	6	8
0–11 years	2	3

*GYN* gynecologist; *OB* obstetrician; *URO* urologist; *US* United States; *UTI* urinary tract infection; *uUTI* uncomplicated urinary tract infection

Values may total more than 100% due to rounding

^a^For US participants only; ^b^Number of patients per month (%) unless otherwise specified

### Physician perception of patient symptom relief, risk factors, recurrent infection rates, and treatment failure rates

**Fig. 1:**
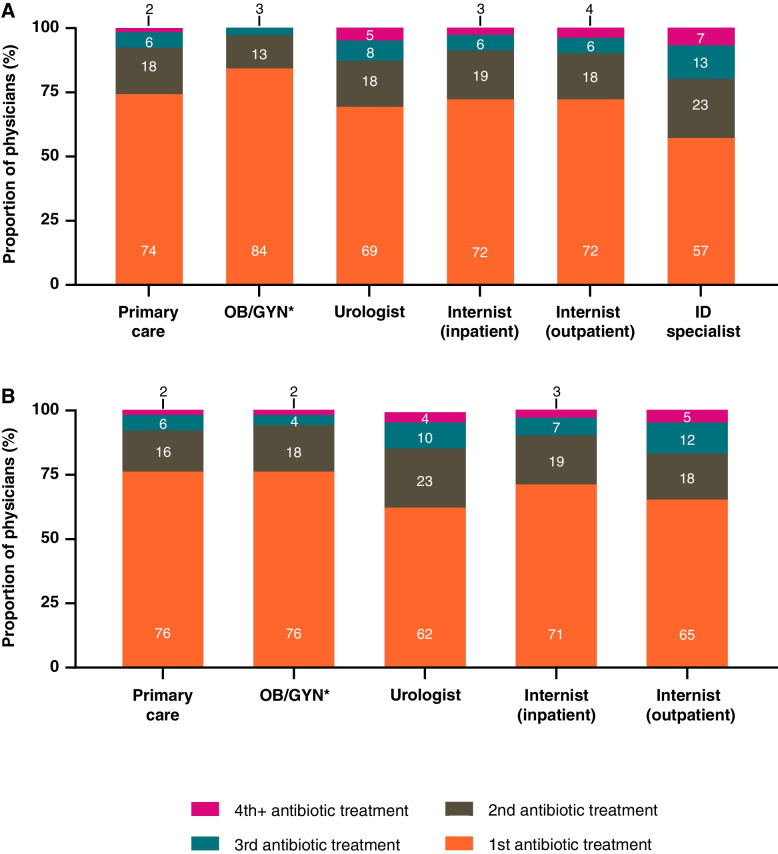
Estimates from (**A**) US and (**B**) German physicians of patients experiencing complete relief from treatment, shown as the percentage of physicians in each specialty and color coded according to the number of antibiotic treatments patients received. *Includes gynecologists, obstetrician/gynecologists and urologist/gynecologists. Percentages may not sum to 100% due to rounding. ID infectious disease; OB/GYN obstetrician/gynecologists; US United States

Across countries and specialties, physicians estimated that 16–43% of patients did not experience complete relief from symptoms after initial treatment (Fig. 1). In both countries, OB/GYNs reported the highest proportion of patients experiencing relief from initial treatment (84% US; 76% Germany) while the lowest estimates came from ID specialists in the US (57%) and urologists in Germany (62%). Among the 5 risk factors queried in the survey, physicians identified post-menopausal women as the highest proportion of their patients with uUTIs (33% US; 30% Germany [Table 2]). Surveyed physicians estimated that approximately one-third of patients experience recurrent uUTI infections. The proportion of patients who experienced treatment failure was estimated to increase with age, with only 3–4% of patients aged 0–11 years failing treatment for uUTI, increasing to 27% for patients aged over 65 years (Table 2). Among several potential clinical factors influencing the likelihood of treatment failure (Table 2), the greatest estimated proportion of patients failing first-line treatment were those with previous confirmed antibiotic-resistant uropathogens, with 29% estimated to fail in the US and 36% in Germany (Table 2).

**Table 2:** Physician perception of patient risk factors, recurrent infection rates and treatment failure

	**US, %** **(*n* = 200)**	**Germany, %** **(*n* = 100)**
**Clinical factor of importance for contracting a uUTI**		
Post-menopausal women	33	30
≥ 65 years of age	28	26
Controlled type II diabetes mellitus	22	20
Moderate chronic kidney disease	17	14
Previous antibiotic resistant uropathogen	17	14
**Proportion of patients experiencing new or recurring infection**		
New infection	67	63
Recurrent infection	33	37
**Proportion of patients per age group that fail treatment**		
> 65 years	27	27
55–65 years	23	21
18–54 years	19	16
12–17 years	6	8
0–11 years	4	3
**Patients with clinical factors that fail first-line therapy**		
Post-menopausal women	18	19
≥ 65 years of age	20	18
Controlled type II diabetes mellitus	18	20
Moderate chronic kidney disease	15	17
Previous antibiotic resistant uropathogen	29	36
**Proportion of patients treated empirically**		
Overall	71	68
General practice, primary care, emergency room	72	70
GYN, OB/GYN, URO/GYN	69	68
URO	58	58
Internist (inpatient)	80	75
Internist (outpatient)	77	80
Infectious disease^a^	80	–
**Average number of patients who respond to empirical treatment**		
Responds	79	80
Requires a change in treatment	21	20

*GYN* gynecologist; *OB* obstetrician; *URO* urologist; *US* United States*; uUTI* uncomplicated urinary tract infection

^a^Infectious disease for US participants only (*n* = 8)

### Diagnosis, disease management, and treatment patterns

**Fig. 2:**
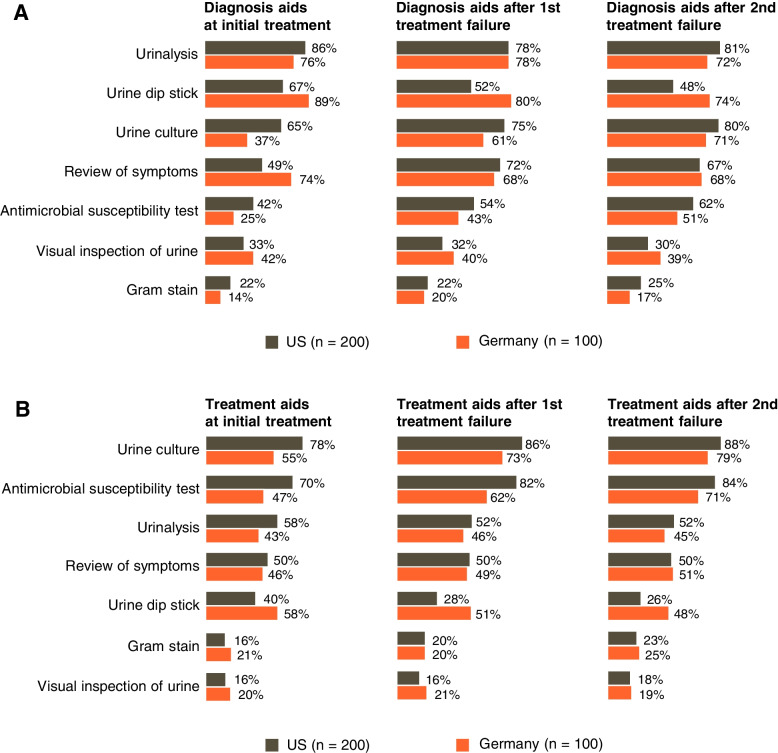
Proportion of physicians in the US (grey) and Germany (orange) reporting use of (**A**) diagnostic aids and (**B**) treatment decision aids for initial and subsequent treatments. Physicians could select multiple responses. US United States

Urinalysis, urine dip stick, and symptom review were used most frequently by surveyed physicians to aid diagnosis, with some variations between countries (Fig. 2A). In the US versus Germany, urinalysis (86% vs. 76%) and urine culture (65% vs. 37%) were used more frequently to aid initial diagnosis; dip stick (67% vs. 89%) and review of symptoms (49% vs. 74%) were used more in Germany. Overall, urine culture and susceptibility testing (CS) were the most commonly used treatment aids (Fig. 2B).

**Fig. 3:**
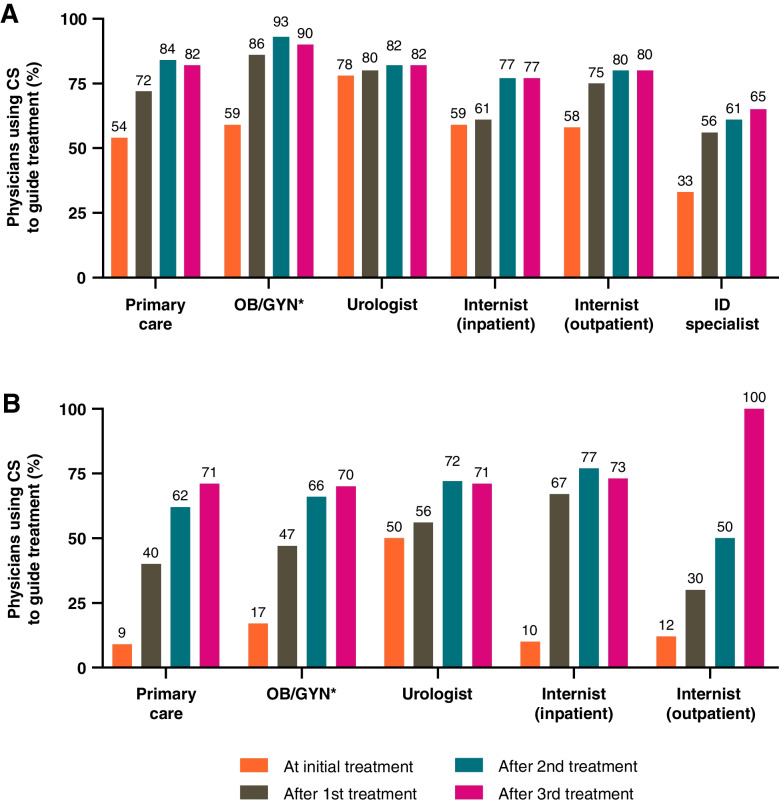
Use of CS testing to guide uUTI treatment in (**A**) the US and (**B**) Germany, initial and subsequent treatments. *Includes gynecologists, obstetrician/gynecologists and urologist/gynecologists. ID infectious disease; CS urine culture and susceptibility; OB/GYN obstetrician/gynecologists; US United States; uUTI uncomplicated urinary tract infection

When assessed by specialty, the use of CS for guiding initial therapy for uUTI differed by physician (range 9–78% [Fig. 3]). CS was more common for initial therapy in the US (range 33–78%) than Germany (range 9–50%), and urologists were the most likely to perform CS for initial therapy (77% US, 50% Germany). The use of CS generally increased with each subsequent treatment required after initial therapy. For a second treatment, CS was used in 30–86% of cases; for a third treatment, CS was used in 50–93% of cases; and for a fourth treatment, CS was used in 65–100% of cases.

**Fig. 4:**
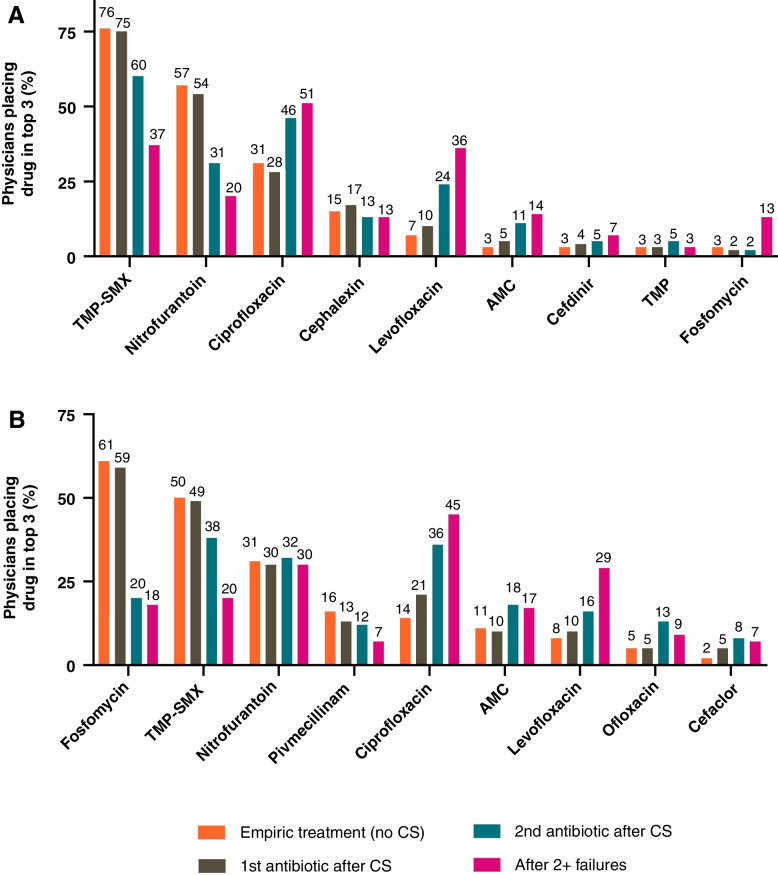
Medication preferences for uUTI by (**A**) US and (**B**) German physicians, initial and subsequent treatments. AMC amoxicillin-clavulanate; CS urine culture and susceptibility; SXT trimethoprim-sulfamethoxazole; TMP trimethoprim; US United States; uUTI uncomplicated urinary tract infection

Physicians estimated that 58–80% of patients were treated empirically, and of these, approximately 80% responded to empiric treatment whilst approximately 20% required a change in treatment (Table 2). Physicians were provided a list of common treatments for uUTI and asked to rank them from most to least commonly prescribed as initial empiric therapy in the last 12 months (Fig. 4). Trimethoprim-sulfamethoxazole (SXT) was reported by US physicians as their preferred first-line medication (76%), followed by nitrofurantoin (57%). German physicians reported fosfomycin as their preferred initial therapy (61%) followed by SXT (50%). In both countries, ciprofloxacin was the preferred treatment after ≥ 2 antibiotic failures (51% US, 45% Germany).

### Treatment satisfaction and goals

A 7-point Likert scale was used to ask physicians their opinion on the current treatment options available for uUTI (1 = extremely disagree, 7 = extremely agree). When asked if they agreed with the statement “I feel there is a good selection of treatment options”, 35% of US and 45% of German physicians agreed (a response of 6 or 7), 4% and 2%, respectively, disagreed (a response of 1 or 2). Similarly, when asked to describe their satisfaction with current treatments (1 = strongly dissatisfied, 7 = strongly satisfied) 50% of US physicians and 57% of German physicians rated the ability of current treatments to provide symptom relief with a 6 or 7. When asked how satisfied they were with the safety of current treatments, 35% of US physicians and 21% of German physicians gave a score of 6 or 7; however, 19% of German physicians gave a score of 1 or 2, while only 6% of US physicians gave this score.

**Fig. 5:**
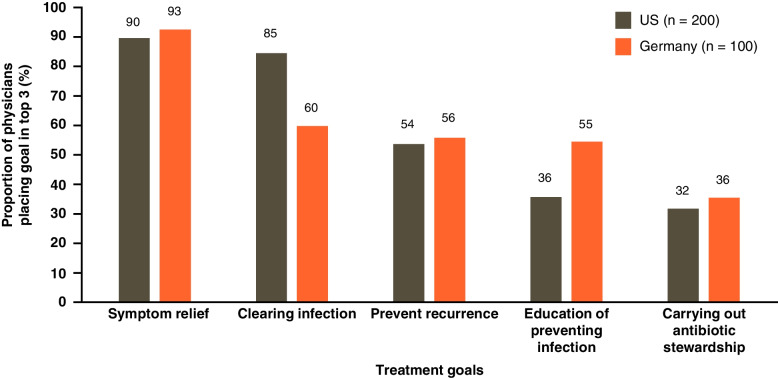
Treatment goal considered in the top 3 most important goals by physicians for managing patients with uUTI. US United States; uUTI uncomplicated urinary tract infection

Physicians were asked to provide their 3 most important treatment goals for uUTI (Fig. 5). More than 90% of physicians across the US and Germany (90% US, 93% Germany) placed relief of symptoms in their top 3 treatment goals. In the US, 85% of physicians placed clearing of infection (microbiological cure) in their top 3 goals, while this was in the top 3 for only 60% of physicians from Germany. Education on preventing infection was more important for German physicians, 55% of whom placed this in their top 3 goals compared with 36% in the US.

### Factors impacting treatment decisions and perceptions of current treatments

The influence of various evidence sources on treatment decisions was assessed by asking physicians to rate the impact that these have on their decisions (1 = no impact at all, 7 = a great deal). Treatment guidelines were reported as greatly influential (score of 6 or 7) on treatment decisions by > 65% of the physicians surveyed (67% US, 66% Germany). US and German physicians reported similar use of national guidelines (62% US, 63% Germany), but local guidelines were used substantially more by US physicians than German (52% vs. 8%) and international guidelines were used more by German physicians than US (48% vs. 10%).

When asked to rate the drugs/drug classes available for the treatment of uUTI (1 = very poor, 7 = exceptional), more than 50% of US physicians gave SXT, NFT, and fluoroquinolones (FQ) a 6 or 7 (58%, 56% and 51%, respectively). German physicians were more conservative, with only fosfomycin being give a 6 or 7 by more than 50% of those surveyed (62%); among German physicians, 31%, 39%, and 30% gave SXT, NFT, and FQ a 6 or 7, respectively. Only 26% of US physicians rated fosfomycin at 6 or 7.

### Symptom and disease burden

**Fig. 6:**
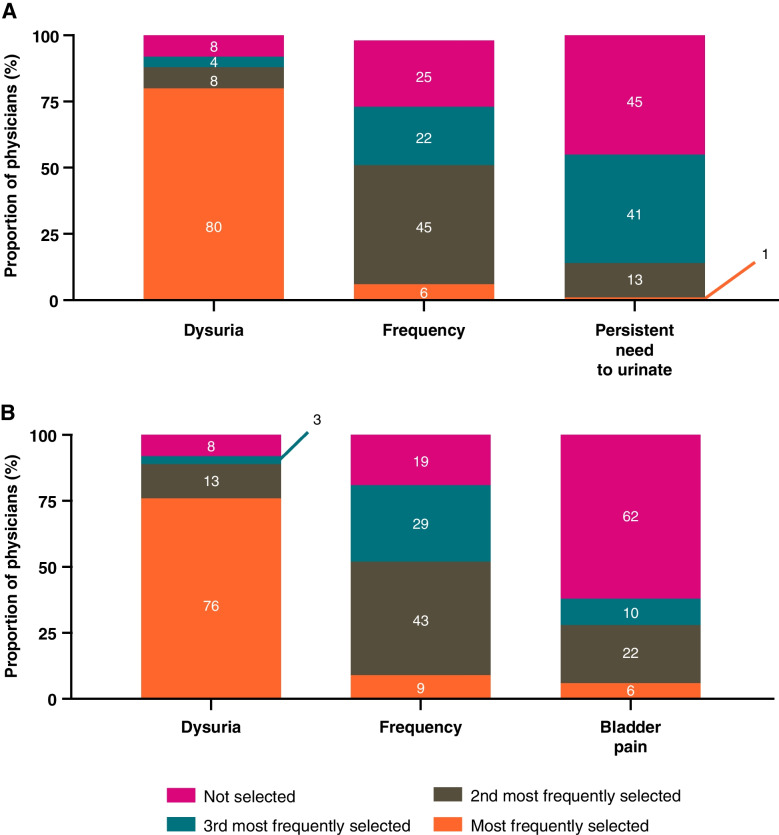
Top 3 most frequently observed symptoms* among patients with uUTI by physicians in (**A**) the US and (**B**) Germany. *Physicians selected symptoms from a list of: dysuria (painful or difficult urination); bladder pain; confusion; tiredness; cloudy urine; blood in urine; need to urinate more frequently than usual; persistent urge to urinate; lower abdominal pain; urge to urinate but can’t; other. Percentages may not sum to 100% due to rounding. US United States; uUTI uncomplicated urinary tract infection

The symptoms observed by physicians in their patients with uUTI were generally similar between the two countries (Fig. 6). Of the top 3 most frequently observed symptoms, dysuria was selected as the most common (80% US, 76% Germany); more frequent urination was selected as the second most common symptom (45% US, 43% Germany).

**Fig. 7:**
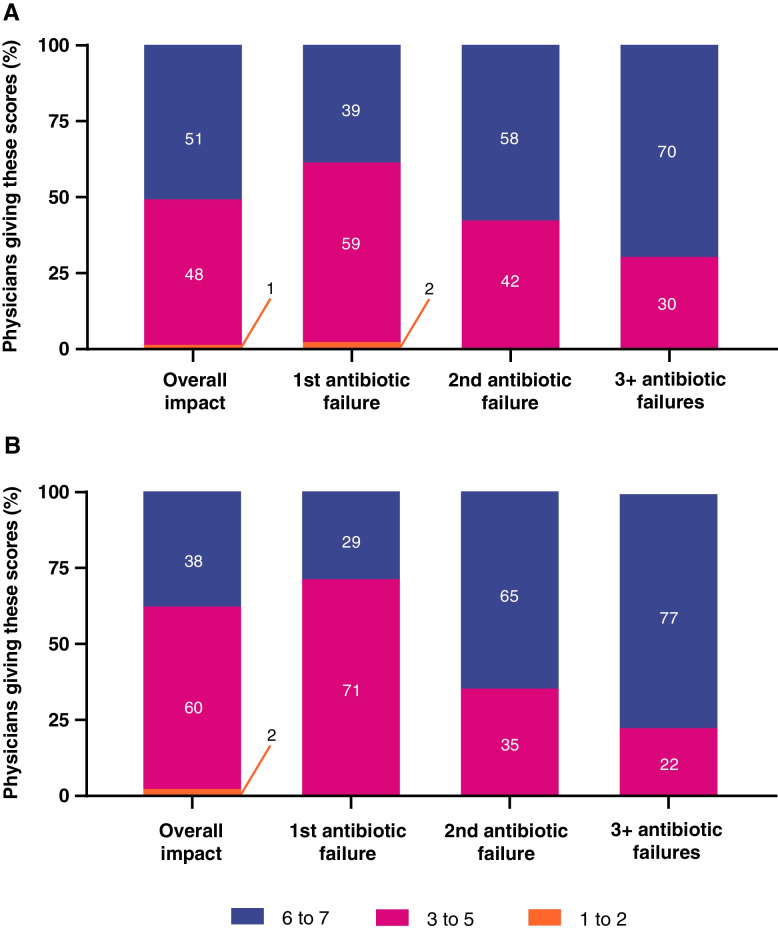
Physicians’ perceptions of the impact of uUTI on patient quality of life in (**A**) the US and (**B**) Germany. Rated on a scale of 1 = “no impact” to 7 = “a great deal of impact”. Percentages may not sum to 100% due to rounding. US United States; uUTI uncomplicated urinary tract infection

**Fig. 8:**
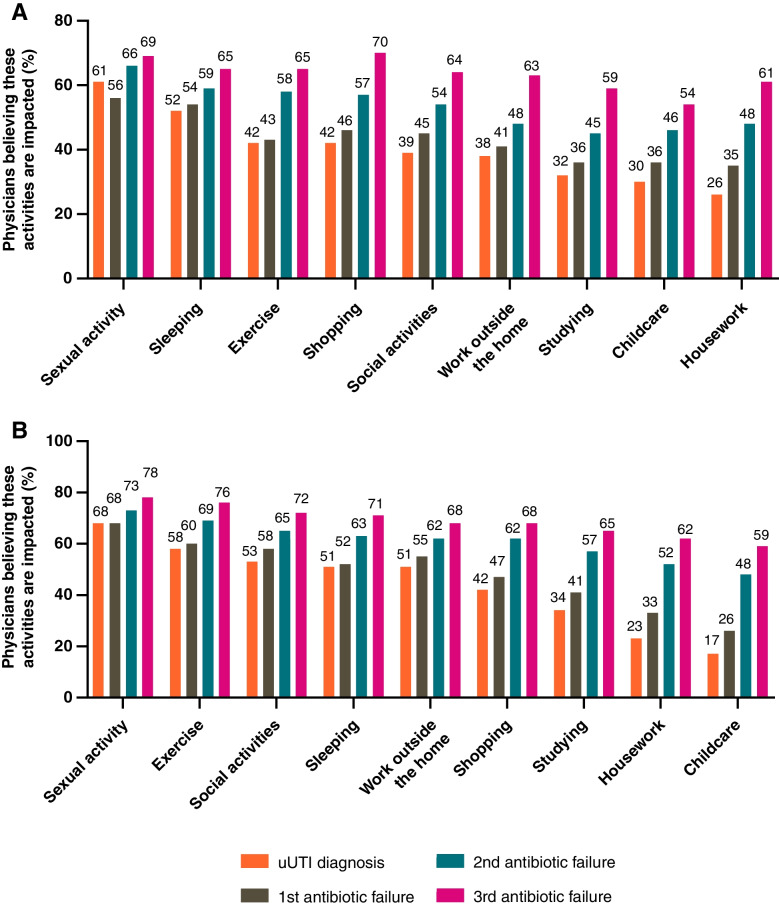
(**A**) US and (**B**) German physicians’ perceptions of the impact of uUTI on patients’ daily activities. Rated on a scale of 1 = “no impact” to 7 = “a great deal of impact”, data shown are the proportions of physicians giving each element a 6 or 7. US United States; uUTI uncomplicated urinary tract infection

In rating the impact that symptoms have on their patients’ quality of life (1 = no impact, 7 = a great deal), 75% of the physicians from each country rated dysuria with a 6 or 7. Persistent urge to urinate was the next most impactful symptom for US physicians with 64% rating this at 6 or 7 (65% Germany), while the next most impactful symptom for German physicians was lower abdomen pain (46% US, 68% Germany). Overall impact of uUTI on patients’ quality of life was scored 6 or 7 (1 = no impact, 7 = a great deal) by 51% of US physicians and 38% of German physicians (Fig. 7); this increased after a second antibiotic failure to 58% and 75%, respectively, and again, after a third antibiotic failure to 70% and 77%, respectively. US and German physicians both estimated that sexual intercourse was the most affected activity among patients following uUTI diagnosis (61% US, 68% Germany). Physicians believed that the impact of uUTI on daily activity increased with each treatment failure (Fig. 8).

### Management of AMR

**Fig. 9:**
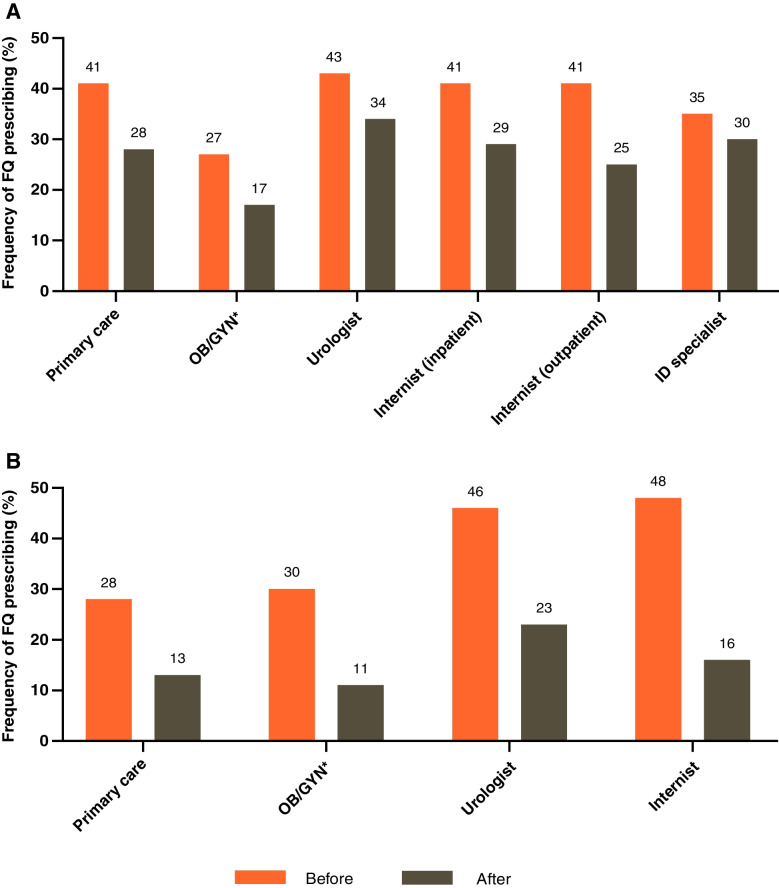
Frequency of FQ prescribing in (**A**) the US and (**B**) Germany before and after new safety warnings. *Includes gynecologists, obstetrician/gynecologists and urologist/gynecologists. FQ fluoroquinolone; ID infectious disease; OB/GYN obstetrician/gynecologists; US United States

Physicians were asked how frequently they prescribed FQ for uUTI before and after the release of new safety concerns for this drug class (Fig. 9). The greatest change was reported by German internists (inpatient) and urologists who reported a decrease in prescribing frequency of 67% and 51%, respectively. Among US physicians, internists in an inpatient setting had the greatest reduction in FQ prescribing (40%), followed by GYN (37%; includes URO/GYN and OB/GYN). Frequency of FQ prescribing post safety announcement was 17–34% in the US and 8–23% in Germany across specialties.

Only 52% of physicians (56% US, 44% Germany) rated their confidence in their knowledge of AMR a 6 or 7 (1 = not at all confident, 7 = a great deal of confidence). Approximately three-quarters of physicians (74% US, 79% Germany) reported discussing AMR with patients. Overall, 68% of physicians (70% US, 68% Germany) considered antibiotic stewardship to be important (score of 6 or 7; 1 = not at all important, 7 = a great deal of importance).

**Fig. 10:**
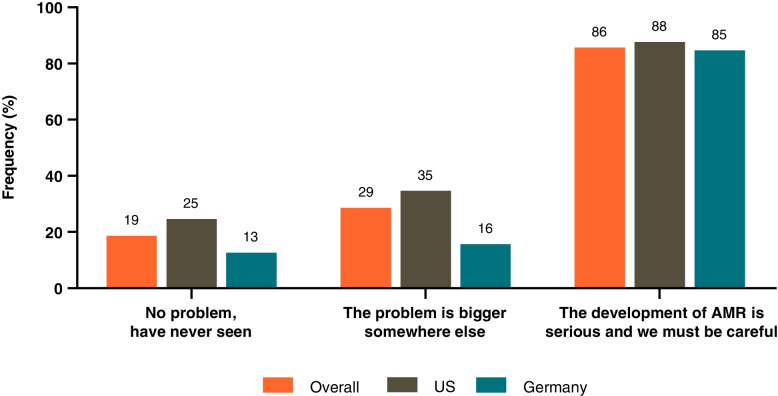
Physicians’ opinions on antimicrobial resistance. Data depicted are the proportions of physicians agreeing with the statements (giving a score of 6 or 7 on the Likert scale). AMR antimicrobial resistance; US United States

The majority of physicians (88% US, 85% Germany) agreed (score 6 or 7) with the Group C statement (“the development of antibiotic resistance is serious and we must be careful”) (Fig. 10). In the US, 35% of physicians also agreed with the Group B statement (“the problem is bigger somewhere else”) and 25% agreed with the group A statement (“no problem with antibiotic resistance, have never seen”) (Fig. 10). Among German physicians, 16% agreed with the Group B statement and 13% with the Group A statement (Fig. 10).

More than 70% of physicians (79% US, 71% Germany) agreed (score of 6 or 7) that a history of AMR in a patient influenced their treatment decisions for that patient. More than half of US physicians (56%) reported having access to local antibiotic resistance data but only 20% of German physicians reported similar access. National data were available to 28% of US physicians and 35% of German physicians. Among the physicians surveyed, 15% from the US and 32% from Germany reported having no access to AMR data (local, regional, or national).

## Discussion

Our survey revealed many similarities between physicians in the US and Germany in their perceptions and management approaches to uUTI. Patients with uUTI comprised approximately 10% of physicians’ workloads across both countries and these patients tended to be between 18 and 54 years of age, reflecting the well-established epidemiology of the condition [7]. Overall, 16–43% of patients were estimated not to receive complete relief from their initial therapy across physician specialties. US and German physicians highlighted post-menopausal women as the clinical factor of greatest importance for contracting a uUTI, and the physicians surveyed estimated that approximately one-third of their patients experience recurrent uUTI. Symptom relief was the primary uUTI treatment goal for US and German physicians. Broadly, physicians recognized that patients are greatly impacted by treatment failures and that antibiotic resistance is a serious problem, though many were not confident in their knowledge on the topic or had insufficient access to relevant information.

Key differences between the country cohorts included their main choice of first-line therapy. While SXT and NFT were popular in both countries, fosfomycin was the most likely to be prescribed for first-line empiric therapy in Germany and least likely in the US for the same indication. To an extent, this difference may be attributable to the recommendations made in different guidelines. For example, the Infectious Diseases Society of America guidance is to prescribe NFT based on its efficacy and relatively low resistance profile, or SXT, which has similar efficacy but generally higher levels of resistance; fosfomycin is listed as an appropriate choice but it is noted that it has lower efficacy than NFT and SXT [2]. In the European Association of Urology (EAU) and German guidelines, however, fosfomycin is listed along with NFT and pivmecillinam (where available) as first-line therapies, with SXT suggested as an alternative where resistance is acceptably low (< 20%) [6, 15]. In both countries, clearing of infection was the second most selected treatment goal; however, 85% of US physicians included it in their top 3 goals and only 60% of German physicians. This suggests that treatment in Germany is more focused on symptom relief than in the US where clearing of infection is also regularly considered.

National guidelines were well used by physicians from both countries, but substantial differences in the use of local and international guidelines were reported. More than half of US physicians used local guidelines while this was true for less than 10% of German physicians. A similar difference was observed for international guidelines, with German physicians reporting greater use than US. These differences may reflect the size and location of the 2 countries. The US covers a larger area, and the prevalence of antibiotic resistance to specific therapies may differ between local regions as a result [16], making local guidelines more important for informing treatment decisions compared to the relatively smaller country of Germany (which is approximately half the size of the state of Texas by area). In addition, due to the movement of people between countries in the European Union that have reciprocal health agreements, physicians in Germany may be more likely to refer to European guidelines than local guidelines. Furthermore, the EAU provides a central source for information in Europe, with regular updates and meetings throughout the year, while there are several organizations in the US offering guidance and information, meaning physicians have more sources to choose from.

The physicians surveyed agreed that dysuria was the most common symptom of uUTI, and 75% (regardless of country) believed that dysuria had a substantial impact on patients’ quality of life; however, half of US physicians and only 38% of German physicians believed that uUTI had a great impact on patients’ lives overall. This finding is important because although previous studies have demonstrated a disconnection between patient and physician perceptions of the impact of uUTI [9], our survey has demonstrated a difference between physicians in different healthcare systems. The caseload of German physicians tended to be higher in our study, potentially reducing consultation times relative to US physicians and leading to differences in perceptions of the impact of uUTI on patients. This could have implications for how physicians choose to treat their patients based on how impactful they believe the infection to be on quality of life.

The introduction of new safety warnings for FQ shortly before the study [17, 18] resulted in a reduced frequency of prescribing for physicians across countries and specialties. There was, however, a notably higher frequency of prescribing among US physicians compared with those in Germany. This was true even when pre-warning levels were similar between the countries (e.g., for urologists and internists). This discrepancy may be related to the number of first-line therapies available to physicians, where pivmecillinam is approved for use in Germany but not the US and fosfomycin use is more prevalent in Germany. Indeed, while approximately 40% of physicians felt a greater selection of treatment options would be beneficial, 45% and 35% of physicians in Germany and the US, respectively, strongly agreed that there is a good selection of treatments available.

The inclusion of physicians from diverse healthcare systems and a range of specialties, giving a broad overview of physician views regarding the treatment of uUTI, was a particular strength of this study. Limitations of the study included the use of self-report data which could be subject to recall-bias. While the data may not accurately reflect some practices (e.g., prescribing patterns), the intention of the study was to gather information on the attitudes and perceptions of physicians, for which self-report is the most appropriate method. The responses of physicians were limited by the questions posed and physicians were often asked to provide their opinions on a list of statements or to select from a list, with no option to provide a free-text answer. The survey was designed to give an overview of opinions and practices most relevant to the treatment of uUTI in the US and Germany. Additionally, the definition of uUTI given to the physicians was different between the 2 countries. The definition provided to German physicians included uncomplicated pyelonephritis per the EAU guidelines [6] while the US definition did not. As such, if physicians in Germany saw a significant number of patients with uncomplicated pyelonephritis, the results could differ on the basis of a different type of infection rather than purely due to differences in management approaches and perceptions. While we did not gather data on the proportion of patients with uncomplicated pyelonephritis treated among the German physicians, we used definitions of uUTI that would be familiar to the physicians, though German guidelines [15] might also have been appropriate in this case but were not considered as the European guidelines were thought to be more widely used and appropriate given the mix of physician specialties included in the survey. Furthermore, among German physicians, fosfomycin, TMP-SMX, nitrofurantoin, and pivmecillinam were reported as the most common antibiotics prescribed in the last 12 months, none of which are indicated for the treatment of uncomplicated pyelonephritis (with the exception of TMP-SMX in the setting of FQ hypersensitivity or resistance) [6]. This suggests that the majority of cases considered by the physicians were of acute cystitis rather than pyelonephritis, but does not guarantee that no cases of pyelonephritis were taken into consideration and this possibility should be understood when interpreting our results. Because of this, we consider our results relevant to the real-world clinical practice of treating uUTI. We also piloted the survey prior to commencing the study to assess its relevance; as such, we believe the most relevant information has been captured.

## Conclusions

For US and German physicians treating patients with uUTI, symptom relief was the primary treatment goal, though many physicians reported that patients do not achieve complete relief from initial therapy. Physicians recognized that uUTIs have a substantial impact on patients’ quality of life and believed that AMR is a serious problem, though many did not have a high level of confidence in their knowledge of this topic. Key differences between US and German physicians included preference of first-line therapy, patient caseload, and use of local versus international guidelines. The goals of physicians in the treatment of patients with uUTI were consistent between countries, though there were nuances to the approaches taken for disease management. These data provide information on differences between healthcare systems that should be considered when developing a new antimicrobial for uUTI; furthermore, it is important to ensure that physicians are well aware of AMR in their region to help inform appropriate treatment strategies.

## Supplementary Information

Additional file 1. Survey Sections.

## Data Availability

The authors confirm that the data supporting the findings of this study are available within the article.

## Abbreviations

AMR: Antimicrobial resistance
CS: Urine culture and susceptibility
EAU: European Association of Urology
FQ: Fluoroquinolones
GYN: Gynecologists
ID: Infectious disease
NFT: Nitrofurantoin
OB: Obstetrician
PCP: Primary care physicians
SXT: Trimethoprim-sulfamethoxazole
URO: Urologists
US: United States
UTI: Urinary tract infection
uUTI: Uncomplicated urinary tract infections
