# Supplementary material for: Unmet needs in uncomplicated urinary tract infection in the United States and Germany: a physician survey
Source: BMC Infect Dis. 2023 May 3;23:281. doi: 10.1186/s12879-023-08207-x (PMC10158246; doi:10.1186/s12879-023-08207-x)
Supplement: Supplementary file 1 — Additional file 1. Survey Sections. [file 12879_2023_8207_MOESM1_ESM.docx]

**Unmet needs in uncomplicated urinary tract infection in the United States and Germany: a physician survey**
V1.0 FINAL

November 2020

**Survey Sections:**

HCP SCREENER

- Caseload
- Primary specialty
- Year qualified
- Location (USA)

WELCOME PAGE AND DISCLAIMER

SECTION A: WORKLOAD CHARACTERISTICS

SECTION B: SYMPTOMS AND BURDEN OF DISEASE

- Regularly reported symptoms
- Impact on QoL
- Most common complications
- Perceived impact on QoL associated with complications
- Ability to conduct daily activities
- Emotional impact of uUTI
- Impact on social activities and work productivity (WPAI)

SECTION C: MANAGEMENT OF DISEASE AND TREATMENT PATTERNS

- Preference for evidence
- Tests conducted for diagnosis and treatment
- Antibiotic awareness
- Influence of antibiotic resistance awareness in decision making

SECTION D: TREATMENT SATISFACTION

- Satisfaction with treatment options
- Reasons for dissatisfaction/satisfaction

SECTION E: BARRIERS AND DRIVERS

- Physician Perception and Brand association
- Rational drivers of treatment use

SECTION F: DEMOGRAPHICS

- Location (Germany and US)
- Care setting

HCP SCREENER

S0. Please indicate the country in which your practice is located:

| 1 | ⭘ | USA |
| --- | --- | --- |
| 2 | ⭘ | Germany |
| 3 | ⭘ | Other <Screen out> |

This study aims to assess the burden of an uncomplicated urinary tract infection uUTI. Note that a complicated urinary tract infection is abbreviated to cUTI.

Definitions:

<USA ONLY>Uncomplicated Urinary Tract Infection(uUTI): A UTI is classified as uncomplicated if there are no functional or anatomical anomalies in the urinary tract, no renal functional impairment, and no concomitant disease that would promote the UTI.

Complicated Urinary Tract Infection (cUTI): A complicated UTI is an infection associated with a condition, such as structural or functional abnormalities of the genitourinary tract or the presence of an underlying disease, which increases the risks of acquiring an infection or of failing therapy.

**<DE ONLY>**

**Uncomplicated Urinary Tract Infection (uUTI):**  Acute, sporadic or recurrent lower (uncomplicated cystitis) and/or upper (uncomplicated pyelonephritis) UTI, limited to non-pregnant, premenopausal women with no known anatomical and functional abnormalities within the urinary tract or comorbidities.

**Complicated Urinary Tract Infection (cUTI):** All UTIs which are not defined as uncomplicated. Meaning in a narrower sense UTIs in a patient with an increased chance of a complicated course: i.e. all men, pregnant women, patients with anatomical or functional abnormalities of the urinary tract, indwelling urinary catheters, renal diseases, and/or with other concomitant immunocompromising diseases for example, diabetes.

S1. Please ***estimate*** your patient caseloads for the following groups **in a typical month (prior to COVID-19)**: <ASK ALL> <s1a should be more than or equal to s1b+s1c >

Please enter an *estimated* number into each column. Don’t worry about providing an exact figure from your records, please just estimate.

|  | a. Your total patient caseload in a typical month | b. Number of complicated UTI patients under your care in a typical month | c. Number of uncomplicated UTI patients under your care in a typical month |
| --- | --- | --- | --- |
| Number of patients: | <SCREEN OUT IF  GPs: BELOW 300  Specialists: BELOW 200>  ________ | ________ | <SCREEN OUT IF BELOW 10 >  __________ |

S2. Are you responsible for making treatment decisions for the uUTI patients under your care? <ASK ALL>

| 1 | ⭘ | Yes |
| --- | --- | --- |
| 2 | ⭘ | No <Screen out> |

S3. In what year did you qualify for your primary specialty? <ASK ALL>

Please select one answer

| 1 | ⭘ | Before 1980 <Screen out> |
| --- | --- | --- |
| 2 | ⭘ | 1980-1990 |
| 3 | ⭘ | 1991-2000 |
| 4 | ⭘ | 2001-2010 |
| 5 | ⭘ | 2011- 2016 |
| 6 | ⭘ | After 2016 <Screen out> |

S4. What is your primary specialty? <ASK ALL>

|  |  | <GERMANY> | <USA> |
| --- | --- | --- | --- |
| 1 | ⭘ | General Practitioner | General Practitioner |
| 2 | ⭘ | Urologist | Urologist |
| 3 | ⭘ | Gynaecologist | Gynaecologist |
| 4 | ⭘ | Urogynecologist | Urogynecologist |
| 5 | ⭘ | Obstetrician-gynaecologist | Obstetrician-gynaecologist |
| 6 | ⭘ | Internist/ Internal medicine specialist | Internist/Internal medicine specialist |
| 7 | ⭘ | Emergency room physician | Emergency Room physician |
| 7 | ⭘ | Infectious diseases specialist | Infectious diseases specialist |
| 8 | ⭘ | Other specialists <Screen out> | Other Specialists<Screen out> |
| 9 | ⭘ | Other <Screen out> | Other <Screen out> |

S5. In which state(s) are you licensed to practice medicine? <ASK US ONLY>

| 1 | ⭘ | Show 46 other states |
| --- | --- | --- |
| 2 | ⭘ | Maine <SCREEN OUT> |
| 3 | ⭘ | Vermont <SCREEN OUT> |
| 4 | ⭘ | Massachusetts <SCREEN OUT> |
| 5 | ⭘ | Minnesota <SCREEN OUT> |

S6. <USA only> In which state(s) do you reside? <ASK ONLY IF S5 IS INDIANA, DISTRICT OF COLUMBIA, OR LOUISIANA>

| 1 | ⭘ | Show 46 other states |
| --- | --- | --- |
| 2 | ⭘ | Maine <SCREEN OUT> |
| 3 | ⭘ | Vermont <SCREEN OUT> |
| 4 | ⭘ | Massachusetts <SCREEN OUT> |
| 5 | ⭘ | Minnesota <SCREEN OUT> |

S7. Are you a state government employee (SGE) or Pharmacy & Therapeutic Committee Members (P&T)? <ASK US ONLY>

| 1 | ⭘ | Yes <Screen out> |
| --- | --- | --- |
| 2 | ⭘ | No |

WELCOME PAGE AND DISCLAIMER

Thank you for completing the screening questions; you are invited to participate in this survey.

This comprises the following elements:

An online **survey** (lasting no longer than 30 minutes), capturing your attitudes and experiences of managing uncomplicated UTIs.

Please click **here** to read the survey’s Informed Consent Form which provides further information on your participation in the survey.

To Programmers: Please refer to separate client Consent Form to be programmed as hyperlink

SECTION A: Workload Characteristics

This section aims to identify the physician workload and characteristics of those patients managed.

Definitions:

<USA ONLY>

Uncomplicated Urinary Tract Infection(uUTI): A UTI is classified as uncomplicated if there are no functional or anatomical anomalies in the urinary tract, no renal functional impairment, and no concomitant disease that would promote the UTI.

Complicated Urinary Tract Infection (cUTI): A complicated UTI is an infection associated with a condition, such as structural or functional abnormalities of the genitourinary tract or the presence of an underlying disease, which increases the risks of acquiring an infection or of failing therapy.

**<DE only>**

**Uncomplicated Urinary Tract Infection(uUTI)**: Acute, sporadic or recurrent lower (uncomplicated cystitis) and/or upper (uncomplicated pyelonephritis) UTI, limited to non-pregnant, premenopausal women with no known anatomical and functional abnormalities within the urinary tract or comorbidities.

**Complicated Urinary Tract Infection(cUTI)**: All UTIs which are not defined as uncomplicated. Meaning in a narrower sense UTIs in a patient with an increased chance of a complicated course: i.e. all men, pregnant women, patients with anatomical or functional abnormalities of the urinary tract, indwelling urinary catheters, renal diseases, and/or with other concomitant immunocompromising diseases for example, diabetes.

AQ1. Please estimate the proportion of your uUTI specific caseload that experience complete relief of symptoms through the: <ASK ALL>

**Definition:** 1^st^ antibiotic treatment may include 1^st^ empiric antibiotic prescription and before completion a change in treatment if culture results require a different antibiotic.

| 1 | __% | 1^st^ antibiotic treatment |
| --- | --- | --- |
| 2 | __% | 2^nd^ antibiotic treatment |
| 3 | __% | 3^rd^ antibiotic treatment |
| 4 | __% | 3+ antibiotic treatments |

AQ2. What proportion of your uUTI patients do you typically prescribe empirically using antibiotic therapy as initial treatment?

| 1 | __% | Patients treated empirically using antibiotic therapy as initial treatment |
| --- | --- | --- |

AQ3. What proportion of your uUTI patients treated with 1^st^ empiric antibiotic treatment typically respond, and following lab results, what proportion of patients need a change in treatment? <ASK ALL>

| 1 | __% | Respond to 1^st^ empiric antibiotic treatment |
| --- | --- | --- |
| 2 | __% | Require change in treatment following lab results |

AQ4a. Earlier you mentioned you see an average of < SCREENER uUTI S1c> uUTI patients each month. On average, what proportion of your patients with uUTIs are new infections and what proportion are recurrent infections? <ASK ALL>

**Definition:** Recurrent UTI: Repeated UTI with a frequency of 2 or more UTIs in the last 6 months or 3 or more UTIs in the last 12 months (*EAU guideline*)

| 1 | __% | New Infections |
| --- | --- | --- |
| 2 | __% | Recurrent infections |

AQ4b. On average, what proportion of your patients with the following classifications typically fail the 1^st^ antibiotic treatment and require a change of treatment?? <ASK ALL>

**Definition:** 1^st^ antibiotic treatment may include 1^st^ empiric antibiotic prescription and a change in treatment before the completion of the initial course if culture results require a different antibiotic.

**Definition:** Failure of treatment defined as: Patient has finished course of antibiotics and still experiencing symptoms or no alleviation of symptoms after the course of treatment has been appropriately completed

| 1 | __% | New Infections |
| --- | --- | --- |
| 2 | __% | Recurrent infections |

AQ5a. What proportion of your patients with uUTIs may be categorised with the following risk factors? <ASK ALL>

| 1 | __% | Post-menopausal women |
| --- | --- | --- |
| 2 | __% | Moderate CKD patients |
| 3 | __% | Previously confirmed antibiotic resistant uropathogen |
| 4 | __% | Controlled Diabetes (T2D) |
| 5 | _% | Elderly =>65 |

AQ5b. What proportion of your uUTI patients with the following risk factors typically fail the 1^st^ antibiotic treatment and require a change of treatment? <ASK ALL>

**Definition:** 1^st^ antibiotic treatment may include 1^st^ empiric antibiotic prescription and a change in treatment before the completion of the initial course if culture results require a different antibiotic.

**Definition:** Failure of treatment defined as: Patient has finished course of antibiotics and still experiencing symptoms or no alleviation of symptoms after the course of treatment has been appropriately completed

| 1 | __% | Post-menopausal women |
| --- | --- | --- |
| 2 | __% | Moderate CKD patients |
| 3 | __% | Previously confirmed antibiotic resistant uropathogen |
| 4 | __% | Controlled Diabetes (T2D) |
| 5 | _% | Elderly =>65 |

AQ6a. Please estimate the proportion of your uUTI specific caseload within the following age groups. <ASK ALL>

| 1 | __% | over 65 years old |
| --- | --- | --- |
| 2 | __% | 55-65 years old |
| 3 | __% | 18-54 years old |
| 4 | __% | 12-17 years old |
| 5 | __% | 0-11 years old |

AQ6b. What proportion of your patients, according to each age group, typically fail the 1^st^ antibiotic treatment and require a change of treatment? <ASK ALL>

**Definition:** Failure of treatment defined as: Patient has finished course of antibiotics and still experiencing symptoms or no alleviation of symptoms after the course of treatment has been appropriately completed.

| 1 | __% | over 65 years old |
| --- | --- | --- |
| 2 | __% | 55-65 years old |
| 3 | __% | 18-54 years old |
| 4 | __% | 12-17 years old |
| 5 | __% | 0-11 years old |

SECTION B: SYMPTOMS and burden of Disease

This section aims to identify the prevalence and impact of symptoms of uncomplicated UTI (uUTIs) on a patient’s quality of life as perceived by the physician. Note that a complicated urinary tract infection is abbreviated to cUTI.

Definitions:

<USA ONLY>

Uncomplicated Urinary Tract Infection(uUTI): A UTI is classified as uncomplicated if there are no functional or anatomical anomalies in the urinary tract, no renal functional impairment, and no concomitant disease that would promote the UTI

Complicated Urinary Tract Infection (cUTI): A complicated UTI is an infection associated with a condition, such as structural or functional abnormalities of the genitourinary tract or the presence of an underlying disease, which increases the risks of acquiring an infection or of failing therapy.

**<DE only>**

**Uncomplicated Urinary Tract Infection(uUTI)**: Acute, sporadic or recurrent lower (uncomplicated cystitis) and/or upper (uncomplicated pyelonephritis) UTI, limited to non-pregnant, premenopausal women with no known anatomical and functional abnormalities within the urinary tract or comorbidities.

**Complicated Urinary Tract Infection(cUTI)**: All UTIs which are not defined as uncomplicated. Meaning in a narrower sense UTIs in a patient with an increased chance of a complicated course: i.e. all men, pregnant women, patients with anatomical or functional abnormalities of the urinary tract, indwelling urinary catheters, renal diseases, and/or with other concomitant immunocompromising diseases for example, diabetes.

BQ1a. From the list below, please indicate the 3 most frequently experienced symptoms by your uUTI patients? <ASK ALL> <SELECT 3 OPTIONS PER COLUMN>

**Please select 3 symptoms, starting with the most bothersome symptom**

|  |  |  |
| --- | --- | --- |
| 1 | Dysuria (painful or difficult urination) | 🞎 |
| 2 | Bladder pain | 🞎 |
| 3 | Confusion | 🞎 |
| 4 | Tiredness | 🞎 |
| 5 | Cloudy urine | 🞎 |
| 6 | Blood in urine | 🞎 |
| 7 | Need to urinate more frequently than usual | 🞎 |
| 8 | Persistent Urge to Urinate | 🞎 |
| 9 | Lower abdominal pain | 🞎 |
| 10 | Urge to urinate but can’t | 🞎 |
| 11 | Other | 🞎 |

BQ1b. Please indicate the level of impact you believe the following symptoms have on your patient’s quality of life.

<ASK ALL><PRESENT ALL>

Please rate the level of impact of the following symptoms on the patient’s quality of life, where 1 = no impact and 7 = a great deal

|  |  | 1 | 2 | 3 | 4 | 5 | 6 | 7 |
| --- | --- | --- | --- | --- | --- | --- | --- | --- |
| 1 | Dysuria (Painful or difficult urination) | ⭘ | ⭘ | ⭘ | ⭘ | ⭘ | ⭘ | ⭘ |
| 2 | Bladder pain | ⭘ | ⭘ | ⭘ | ⭘ | ⭘ | ⭘ | ⭘ |
| 3 | Confusion | ⭘ | ⭘ | ⭘ | ⭘ | ⭘ | ⭘ | ⭘ |
| 4 | Tiredness | ⭘ | ⭘ | ⭘ | ⭘ | ⭘ | ⭘ | ⭘ |
| 5 | Cloudy urine | ⭘ | ⭘ | ⭘ | ⭘ | ⭘ | ⭘ | ⭘ |
| 6 | Blood in urine | ⭘ | ⭘ | ⭘ | ⭘ | ⭘ | ⭘ | ⭘ |
| 7 | Need to urinate more frequently than usual | ⭘ | ⭘ | ⭘ | ⭘ | ⭘ | ⭘ | ⭘ |
| 8 | Persistent urge to urinate | ⭘ | ⭘ | ⭘ | ⭘ | ⭘ | ⭘ | ⭘ |
| 9 | Lower abdominal pain | ⭘ | ⭘ | ⭘ | ⭘ | ⭘ | ⭘ | ⭘ |
| 10 | Urge to urinate but can’t | ⭘ | ⭘ | ⭘ | ⭘ | ⭘ | ⭘ | ⭘ |
| 11 | Other | ⭘ | ⭘ | ⭘ | ⭘ | ⭘ | ⭘ | ⭘ |

BQ2a. If left untreated or inappropriately treated, what 3 complications do your uUTI patients most frequently present with? <ASK ALL>

**Please select 3 complications, starting with the most frequent.**

| 1 | Reduced kidney function | 🞎 |
| --- | --- | --- |
| 2 | Complicated Pyelonephritis | 🞎 |
| 3 | Acute Pyelonephritis / Acute uncomplicated Pyelonephritis | 🞎 |
| 4 | Severe confusion <US only> | 🞎 |
| 5 | Chronic UTI | 🞎 |
| 6 | Recurrent UTI | 🞎 |
| 7 | Sepsis | 🞎 |
| 8 | Bacteremia | 🞎 |
| 9 | Opportunistic/ secondary infections | 🞎 |
| 10 | Other | 🞎 |

BQ2b. What level of impact do the following complications have on your patient’s quality of life?

<PRESENT ALL OPTIONS>

Please rate the impact of the following complications on a patient’s quality of life, where 1 = no impact and 7 = a great deal

|  |  | 1 | 2 | 3 | 4 | 5 | 6 | 7 |
| --- | --- | --- | --- | --- | --- | --- | --- | --- |
| 1 | Reduced kidney function | ⭘ | ⭘ | ⭘ | ⭘ | ⭘ | ⭘ | ⭘ |
| 2 | Complicated Pyelonephritis | ⭘ | ⭘ | ⭘ | ⭘ | ⭘ | ⭘ | ⭘ |
| 3 | Acute Pyelonephritis | ⭘ | ⭘ | ⭘ | ⭘ | ⭘ | ⭘ | ⭘ |
| 4 | Severe confusion [US only] | ⭘ | ⭘ | ⭘ | ⭘ | ⭘ | ⭘ | ⭘ |
| 5 | Chronic UTI | ⭘ | ⭘ | ⭘ | ⭘ | ⭘ | ⭘ | ⭘ |
| 6 | Recurrent UTI | ⭘ | ⭘ | ⭘ | ⭘ | ⭘ | ⭘ | ⭘ |
| 7 | Sepsis | ⭘ | ⭘ | ⭘ | ⭘ | ⭘ | ⭘ | ⭘ |
| 8 | Bacteremia | ⭘ | ⭘ | ⭘ | ⭘ | ⭘ | ⭘ | ⭘ |
| 9 | Opportunistic/ secondary infections | ⭘ | ⭘ | ⭘ | ⭘ | ⭘ | ⭘ | ⭘ |
| 10 | Other | ⭘ | ⭘ | ⭘ | ⭘ | ⭘ | ⭘ | ⭘ |

BQ3a. Overall, based on your experience, to what extent do you believe uUTIs affect a patient’s quality of life?

Please rate the statement below, where 1 = no impact and 7 = a great deal.

| 1 | 2 | 3 | 4 | 5 | 6 | 7 |
| --- | --- | --- | --- | --- | --- | --- |
| ⭘ | ⭘ | ⭘ | ⭘ | ⭘ | ⭘ | ⭘ |

BQ3b. Overall, based on your experience, to what extent do you believe failure to treat uUTI’s with the < 1^st^ /2^nd^ / 3^rd^ or subsequent antibiotic treatment > affects a patient’s quality of life? <ASK ALL>

**Definition:** Failure of treatment defined as: Patient has finished course of antibiotics and still experiencing symptoms or no alleviation of symptoms after the course of treatment has been appropriately completed.

**Definition:** 1^st^antibiotic treatment may include 1^st^ empiric antibiotic treatment and a change in treatment before the completion of the initial course if culture results require a different antibiotic.

Please rate the statement below, where 1 = no impact and 7 = a great deal.

|  |  | Impact on patient QoL having failed <1^st^/2^nd^/3^rd^ antibiotic or subsequent > | | | | | | |
| --- | --- | --- | --- | --- | --- | --- | --- | --- |
|  |  | 1 | 2 | 3 | 4 | 5 | 6 | 7 |
| 1 | 1^st^ antibiotic treatment failure | ⭘ | ⭘ | ⭘ | ⭘ | ⭘ | ⭘ | ⭘ |
| 2 | 2^nd^ antibiotic treatment failure | ⭘ | ⭘ | ⭘ | ⭘ | ⭘ | ⭘ | ⭘ |
| 3 | 3^rd^ antibiotic treatment failure | ⭘ | ⭘ | ⭘ | ⭘ | ⭘ | ⭘ | ⭘ |

BQ4a. To what extent do the typical symptoms reported by an uUTI patient affect their ability to conduct the following daily activities? <ASK ALL>

Please rate the impact typical symptoms have on each activity below, where 1 = no impact at all and 7 = a great deal.

|  |  | 1 | 2 | 3 | 4 | 5 | 6 | 7 |
| --- | --- | --- | --- | --- | --- | --- | --- | --- |
| 1 | Shopping/running errands | ⭘ | ⭘ | ⭘ | ⭘ | ⭘ | ⭘ | ⭘ |
| 2 | Childcare | ⭘ | ⭘ | ⭘ | ⭘ | ⭘ | ⭘ | ⭘ |
| 3 | Housework/chores | ⭘ | ⭘ | ⭘ | ⭘ | ⭘ | ⭘ | ⭘ |
| 4 | Exercise | ⭘ | ⭘ | ⭘ | ⭘ | ⭘ | ⭘ | ⭘ |
| 5 | School/studying | ⭘ | ⭘ | ⭘ | ⭘ | ⭘ | ⭘ | ⭘ |
| 6 | Sleeping | ⭘ | ⭘ | ⭘ | ⭘ | ⭘ | ⭘ | ⭘ |
| 7 | Social activities | ⭘ | ⭘ | ⭘ | ⭘ | ⭘ | ⭘ | ⭘ |
| 8 | Work outside the home | ⭘ | ⭘ | ⭘ | ⭘ | ⭘ | ⭘ | ⭘ |
| 9 | Sexual Activity (Only consider patients within the legal age of consent) | ⭘ | ⭘ | ⭘ | ⭘ | ⭘ | ⭘ | ⭘ |

BQ4b. For those patients who fail their 1^st^ antibiotic treatment or subsequent treatments, to what extent do the typical symptoms experienced by an uUTI patient affect their ability to conduct the following daily activities? <ASK ALL><REPEAT FOR <1^st^/2^nd^/3^rd^ antibiotic or subsequent >

Please rate the statement below, where 1 = no impact at all and 7 = a great deal.

|  |  | Ability to conduct the daily activities having failed <1^st^/2^nd^/3^rd^ antibiotic or subsequent > | | | | | | |
| --- | --- | --- | --- | --- | --- | --- | --- | --- |
|  |  | 1 | 2 | 3 | 4 | 5 | 6 | 7 |
| 1 | Shopping/running errands | ⭘ | ⭘ | ⭘ | ⭘ | ⭘ | ⭘ | ⭘ |
| 2 | Childcare | ⭘ | ⭘ | ⭘ | ⭘ | ⭘ | ⭘ | ⭘ |
| 3 | Housework/chores | ⭘ | ⭘ | ⭘ | ⭘ | ⭘ | ⭘ | ⭘ |
| 4 | Exercise | ⭘ | ⭘ | ⭘ | ⭘ | ⭘ | ⭘ | ⭘ |
| 5 | School/studying | ⭘ | ⭘ | ⭘ | ⭘ | ⭘ | ⭘ | ⭘ |
| 6 | Sleeping | ⭘ | ⭘ | ⭘ | ⭘ | ⭘ | ⭘ | ⭘ |
| 7 | Social activities | ⭘ | ⭘ | ⭘ | ⭘ | ⭘ | ⭘ | ⭘ |
| 8 | Work outside the home | ⭘ | ⭘ | ⭘ | ⭘ | ⭘ | ⭘ | ⭘ |
| 9 | Sexual Activity (Only consider patients within the legal age of consent) | ⭘ | ⭘ | ⭘ | ⭘ | ⭘ | ⭘ | ⭘ |

BQ5a. In your opinion, to what extent do you think patients may feel/experience the following as a result of their uUTI? <ASK ALL>

Please rate the statement below, where 1 = no impact at all and 7 = a great deal.

|  |  | 1 | 2 | 3 | 4 | 5 | 6 | 7 |
| --- | --- | --- | --- | --- | --- | --- | --- | --- |
| 1 | Anxious/nervous | ⭘ | ⭘ | ⭘ | ⭘ | ⭘ | ⭘ | ⭘ |
| 2 | Depressed | ⭘ | ⭘ | ⭘ | ⭘ | ⭘ | ⭘ | ⭘ |
| 3 | Frustration at failure of previous treatment | ⭘ | ⭘ | ⭘ | ⭘ | ⭘ | ⭘ | ⭘ |
| 4 | Worry that their symptoms will get worse | ⭘ | ⭘ | ⭘ | ⭘ | ⭘ | ⭘ | ⭘ |
| 5 | Stress | ⭘ | ⭘ | ⭘ | ⭘ | ⭘ | ⭘ | ⭘ |
| 6 | Helpless feeling | ⭘ | ⭘ | ⭘ | ⭘ | ⭘ | ⭘ | ⭘ |
| 7 | Social isolation | ⭘ | ⭘ | ⭘ | ⭘ | ⭘ | ⭘ | ⭘ |
| 8 | Unable to do the things they enjoy (e.g. go out for dinner, to the gym etc) | ⭘ | ⭘ | ⭘ | ⭘ | ⭘ | ⭘ | ⭘ |
| 9 | Change in emotional well being | ⭘ | ⭘ | ⭘ | ⭘ | ⭘ | ⭘ | ⭘ |

BQ5b. For those patients who fail their 1^st^ antibiotic treatment or subsequent treatments, to what extent do you think patients may feel/experience the following as a result of their uUTI? <ASK ALL> <REPEAT FOR<1^st^/2^nd^/3^rd^ antibiotic or subsequent >

Please rate the statement below, where 1 = no impact at all and 7 = a great deal.

|  |  | Level to which patients may feel/experience the following having failed <1^st^/2^nd^/3^rd^ antibiotic or subsequent >: | | | | | | |
| --- | --- | --- | --- | --- | --- | --- | --- | --- |
|  |  | 1 | 2 | 3 | 4 | 5 | 6 | 7 |
| 1 | Anxious/nervous | ⭘ | ⭘ | ⭘ | ⭘ | ⭘ | ⭘ | ⭘ |
| 2 | Depressed | ⭘ | ⭘ | ⭘ | ⭘ | ⭘ | ⭘ | ⭘ |
| 3 | Frustration at failure of previous treatment | ⭘ | ⭘ | ⭘ | ⭘ | ⭘ | ⭘ | ⭘ |
| 4 | Worry that their symptoms will get worse | ⭘ | ⭘ | ⭘ | ⭘ | ⭘ | ⭘ | ⭘ |
| 5 | Stress | ⭘ | ⭘ | ⭘ | ⭘ | ⭘ | ⭘ | ⭘ |
| 6 | Helpless feeling | ⭘ | ⭘ | ⭘ | ⭘ | ⭘ | ⭘ | ⭘ |
| 7 | Social isolation | ⭘ | ⭘ | ⭘ | ⭘ | ⭘ | ⭘ | ⭘ |
| 8 | Unable to do the things they enjoy (e.g. go out for dinner, to the gym etc) | ⭘ | ⭘ | ⭘ | ⭘ | ⭘ | ⭘ | ⭘ |
| 9 | Change in emotional wellbeing | ⭘ | ⭘ | ⭘ | ⭘ | ⭘ | ⭘ | ⭘ |

BQ6. For those recurrent UTI patients who fail their 1^st^ line of antibiotic treatments or subsequent treatments, to what extent do you think patients may feel/experience the following as a result of their recurrent uUTI? <ASK ALL> <REPEAT FOR<1^st^/2^nd^/3^rd^ antibiotic or subsequent >

**Definition:** Recurrent UTI defined as: Repeated UTI with a frequency of 2 or more UTIs in the last 6 months or 3 or more UTIs in the last 12 months.

Please rate the statement below, where 1 = no impact at all and 7 = a great deal.

|  |  | Level to which patients may feel/experience the following having failed <1^st^/2^nd^/3^rd^ antibiotic or subsequent >: | | | | | | |
| --- | --- | --- | --- | --- | --- | --- | --- | --- |
|  |  | 1 | 2 | 3 | 4 | 5 | 6 | 7 |
| 1 | Anxious/nervous | ⭘ | ⭘ | ⭘ | ⭘ | ⭘ | ⭘ | ⭘ |
| 2 | Depressed | ⭘ | ⭘ | ⭘ | ⭘ | ⭘ | ⭘ | ⭘ |
| 3 | Frustration at failure of previous treatment | ⭘ | ⭘ | ⭘ | ⭘ | ⭘ | ⭘ | ⭘ |
| 4 | Worry that their symptoms will get worse | ⭘ | ⭘ | ⭘ | ⭘ | ⭘ | ⭘ | ⭘ |
| 5 | Stress | ⭘ | ⭘ | ⭘ | ⭘ | ⭘ | ⭘ | ⭘ |
| 6 | Helpless feeling | ⭘ | ⭘ | ⭘ | ⭘ | ⭘ | ⭘ | ⭘ |
| 7 | Social isolation | ⭘ | ⭘ | ⭘ | ⭘ | ⭘ | ⭘ | ⭘ |
| 8 | Unable to do the things they enjoy (e.g. go out for dinner, to the gym etc) | ⭘ | ⭘ | ⭘ | ⭘ | ⭘ | ⭘ | ⭘ |
| 9 | Change in emotional wellbeing | ⭘ | ⭘ | ⭘ | ⭘ | ⭘ | ⭘ | ⭘ |

BQ7a.In your opinion, to what extent do you believe uUTIs impact the following aspects of a patient’s life? <ASK ALL>

Please rate the statement below, where 1 = no impact at all and 7 = a great deal.

|  |  | 1 | 2 | 3 | 4 | 5 | 6 | 7 |
| --- | --- | --- | --- | --- | --- | --- | --- | --- |
| 1 | Cut down on the amount of time spent on work or other activities | ⭘ | ⭘ | ⭘ | ⭘ | ⭘ | ⭘ | ⭘ |
| 2 | Accomplished less than they would like | ⭘ | ⭘ | ⭘ | ⭘ | ⭘ | ⭘ | ⭘ |
| 3 | Limited in the kind of work or other activities | ⭘ | ⭘ | ⭘ | ⭘ | ⭘ | ⭘ | ⭘ |
| 4 | Had difficulty performing work or other activities (for example, it took extra effort) | ⭘ | ⭘ | ⭘ | ⭘ | ⭘ | ⭘ | ⭘ |
| 5 | Interfered with their social activities (like visiting friends, relatives, etc.) | ⭘ | ⭘ | ⭘ | ⭘ | ⭘ | ⭘ | ⭘ |
| 6 | Financial burden  (out of pocket expense) | ⭘ | ⭘ | ⭘ | ⭘ | ⭘ | ⭘ | ⭘ |
| 7 | Caregiver burden | ⭘ | ⭘ | ⭘ | ⭘ | ⭘ | ⭘ | ⭘ |

BQ7b. For those patients who fail their 1^st^ antibiotic treatment or subsequent treatments, to what extent do you believe uUTIs impact the following aspects of a patient’s life? <ASK ALL><REPEAT FOR<1^st^/2^nd^/3^rd^ antibiotic or subsequent >

Please rate the statement below, where 1 = no impact at all and 7 = a great deal.

|  |  | Impact on aspects of patient’s life having failed <1^st^/2^nd^/3^rd^ antibiotic or subsequent >: | | | | | | |
| --- | --- | --- | --- | --- | --- | --- | --- | --- |
|  |  | 1 | 2 | 3 | 4 | 5 | 6 | 7 |
| 1 | Cut down on the amount of time spent on work or other activities | ⭘ | ⭘ | ⭘ | ⭘ | ⭘ | ⭘ | ⭘ |
| 2 | Accomplished less than they would like | ⭘ | ⭘ | ⭘ | ⭘ | ⭘ | ⭘ | ⭘ |
| 3 | Limited in the kind of work or other activities | ⭘ | ⭘ | ⭘ | ⭘ | ⭘ | ⭘ | ⭘ |
| 4 | Had difficulty performing work or other activities (for example, it took extra effort) | ⭘ | ⭘ | ⭘ | ⭘ | ⭘ | ⭘ | ⭘ |
| 5 | Interfered with their social activities (like visiting friends, relatives, etc.) | ⭘ | ⭘ | ⭘ | ⭘ | ⭘ | ⭘ | ⭘ |
| 6 | Financial burden  (out of pocket expense) | ⭘ | ⭘ | ⭘ | ⭘ | ⭘ | ⭘ | ⭘ |
| 7 | Caregiver burden | ⭘ | ⭘ | ⭘ | ⭘ | ⭘ | ⭘ | ⭘ |

BQ8. Typically, after initial treatment, what do you do if a patient returns with symptoms within 28 days of the initial episode? **Select all that apply** <ASK ALL>

| 1 | 🞎 | Prolong treatment duration of the previous antibiotic |
| --- | --- | --- |
| 2 | 🞎 | Perform a susceptibility test to guide second treatment |
| 3 | 🞎 | Empirically prescribe a different antibiotic |
| 4 | 🞎 | Perform culture and susceptibility test to identify new infection |
| 5 | 🞎 | Refer to specialist <PRIMARY CARE PHYSICIAN ONLY> |
| 6 | 🞎 | Refer to other specialist <SPECIALISTS ONLY> |

BQ9. For those patients who fail to respond to treatment, what is the typical time taken between the start of the 1^st^ antibiotic treatment to the start of the 2^nd^ antibiotic treatment? <ASK ALL> REPEAT FOR 2^nd^ AND 3^rd^ ANTIBIOTIC TREATMENTs

|  | Time from 1^st^ 🡪 2^nd^ antibiotic treatment | Time from 2^nd^ 🡪 3^rd^ antibiotic treatment | Time from 3^rd^ 🡪 4^th^ antibiotic treatment |
| --- | --- | --- | --- |
| 1 | __Days (max. 28 days) | __Days (max. 28 days) | __Days (max. 28 days) |

BQ10. Typically, how many visits would a uUTI patient treated with the <1^st^ / 2^nd^ / 3^rd^ > antibiotic treatment attend with the following physician types for the management of their condition? <ASK ALL> REPEAT FOR 2^nd^ AND 3^rd^ ANTIBIOTIC TREATMENTS

|  |  | 1^st^ antibiotic treatment | Failed 1^st^ line and receiving 2^nd^ antibiotic treatment | Failed 2^nd^ line and receiving 3^rd^+ antibiotic treatment |
| --- | --- | --- | --- | --- |
| 1 | Frequency of visit to GP/PCP/Internist | __Visits | __Visits | __Visits |
| 2 | Frequency of visit to Specialist | __Visits | __Visits | __Visits |

BQ11. Typically, at which line of treatment would you refer a patient to a specialist for the management of their uUTI? <ASK TO PRIMARY CARE PHYSICIANS ONLY>

| 1 | ⭘ | Would not typically refer |
| --- | --- | --- |
| 2 | ⭘ | 1^st^ antibiotic treatment |
| 3 | ⭘ | 2^nd^ antibiotic treatment |
| 4 | ⭘ | 3^rd^ antibiotic treatment or later |

SECTION C: MANAGEMENT OF DISEASE AND TREATMENT PATTERNS

Section C focuses on the treatment options currently available for physicians and influence on the physician’s treatment practices including evidence preference, diagnosis tests and antibiotic resistance awareness.

These questions are focused on the typical treatment and management of uUTIs before the COVID-19 pandemic, therefore please answer considering a typical month.

CQ1. To what extent does each of the following sources of evidence influence your treatment decisions? <ASK ALL>

**Please rate the source of evidence below, where 1 = no impact at all and 7 = a great deal**

|  |  | 1 | 2 | 3 | 4 | 5 | 6 | 7 |
| --- | --- | --- | --- | --- | --- | --- | --- | --- |
| 1 | Real World Evidence | ⭘ | ⭘ | ⭘ | ⭘ | ⭘ | ⭘ | ⭘ |
| 2 | Patient Outcome Data | ⭘ | ⭘ | ⭘ | ⭘ | ⭘ | ⭘ | ⭘ |
| 3 | Clinical Trial Data | ⭘ | ⭘ | ⭘ | ⭘ | ⭘ | ⭘ | ⭘ |
| 4 | Treatment Guidelines | ⭘ | ⭘ | ⭘ | ⭘ | ⭘ | ⭘ | ⭘ |
| 5 | Health Economic data | ⭘ | ⭘ | ⭘ | ⭘ | ⭘ | ⭘ | ⭘ |
| 6 | Clinical Care Protocols | ⭘ | ⭘ | ⭘ | ⭘ | ⭘ | ⭘ | ⭘ |

CQ2. In what proportion of cases do you perform culture and susceptibility testing for selection of treatment: <ASK ALL>

| 1 | __% | For initial antibiotic treatment |
| --- | --- | --- |
| 2 | __% | After 1^st^ antibiotic treatment |
| 3 | __% | After 2^nd^ antibiotic treatment |
| 4 | __% | After 3^rd^ antibiotic treatments |

CQ3a. Which of the following do you typically use to aid diagnosis and treatment choice at first visit? <ASK ALL>

**Select all that apply**

|  |  | Diagnosis | Treatment |
| --- | --- | --- | --- |
| 1 | Urinalysis | 🞎 | 🞎 |
| 2 | Urine dip stick | 🞎 | 🞎 |
| 3 | Gram Stain | 🞎 | 🞎 |
| 4 | Urine culture | 🞎 | 🞎 |
| 5 | Antimicrobial susceptibility test | 🞎 | 🞎 |
| 6 | Visual inspection of urine | 🞎 | 🞎 |
| 7 | Review of patient reported symptoms | 🞎 | 🞎 |
| 8 | Other | 🞎 | 🞎 |

CQ3b. Which of the following do you typically use to aid in diagnosis and or treatment choice at the first follow up visit if the patient has a treatment failure with initial antibiotic prescribed? <ASK ALL>

**Select all that apply**

|  |  | Diagnosis | Treatment |
| --- | --- | --- | --- |
| 1 | Urinalysis | 🞎 | 🞎 |
| 2 | Urine dip stick | 🞎 | 🞎 |
| 3 | Gram stain | 🞎 | 🞎 |
| 4 | Urine culture | 🞎 | 🞎 |
| 5 | Antimicrobial Susceptibility test | 🞎 | 🞎 |
| 6 | Visual inspection of urine | 🞎 | 🞎 |
| 7 | Review of patient reported symptoms | 🞎 | 🞎 |
| 8 | Other | 🞎 | 🞎 |

CQ3c. Which of the following do you typically use to aid in diagnosis and or treatment choice at the second follow-up visit if the patient has a treatment failure with the second antibiotic prescribed? <ASK ALL>

**Select all that apply**

|  |  | Diagnosis | Treatment |
| --- | --- | --- | --- |
| 1 | Urinalysis | 🞎 | 🞎 |
| 2 | Urine dip stick | 🞎 | 🞎 |
| 3 | Gram Stain | 🞎 | 🞎 |
| 4 | Urine culture | 🞎 | 🞎 |
| 5 | Antimicrobial Susceptibility test | 🞎 | 🞎 |
| 6 | Visual inspection of urine | 🞎 | 🞎 |
| 7 | Review of patient reported symptoms | 🞎 | 🞎 |
| 8 | Other | 🞎 | 🞎 |

CQ4. Listed below are a number of antibiotic therapies associated with the treatment of uUTI:

1. Please rank, by selecting most to least common, the overall medication from the last 12 months you prescribed as empiric initial antibiotic treatment for uUTI patients without previous lab results (culture and sensitivity)? <ASK ALL>
2. Please rank, by selecting most to least common, the overall medication from the last 12 months you prescribed as first antibiotic treatment for uUTI patients who had lab results available? <ASK ALL>
3. Please rank, by selecting most to least common, the overall medication from the last 12 months you prescribed as the second antibiotic treatment for uUTI patients who had lab results available? <ASK ALL>
4. Please rank, by selecting most to least common, the overall medication from the last 12 months you prescribed following failure of two or more antibiotic treatments for uUTI patients who had lab results available? <ASK ALL>

**Select the most common to least common treatment by checking the box, if you do not use a medication leave it unselected.**

|  |  | a) | b) | c) |
| --- | --- | --- | --- | --- |
| 1. | [US only]Proloprim, Monotrim, Triprim (trimethoprim) | 🞎 | 🞎 | 🞎 |
| 2. | [US Only] Bactrim, Bactrim DS, Co-trimoxazole Septra, Septra DS, Sulfamethoprim, Sulfamethoprim-DS, Sulmeprimy, Sulfatrim, Sulfatrim Pediatric, Sulfatrim-DS, Sulfatrim-SS, Sulmeprim, Sulmeprim Pediatric, Mecenellam, Trimethorprim, Uroplus DS, Uroplus SS, (trimethoprim-sulfamethoxazole)  [DE Only] Bactrim, Bactrim DS, Bactoreduct, Duratrimet, Eusaprim, Kepinol, Microtrim, Omsat, Sigaprim, Cotrimoxazole (trimethoprim-sulfamethoxazole) | 🞎 | 🞎 | 🞎 |
| 3. | [US Only] Furadantin, Furalan, Ivadantin, Macrobid, Macrodantin (nitrofurantoin)  [DE Only] Furadantin retard, Furadantin RP, Nifurantin, Nifuretten, Nitrofurantoin-ratiopharm, Ituran (nitrofurantoin) | 🞎 | 🞎 | 🞎 |
| 4. | [US Only] Monurol, (Fosfomycin trometamol)  [DE only] Infectofos (Fosfomycin trometamol) | 🞎 | 🞎 | 🞎 |
| 5. | [US Only] Cipro, Cipro XR, Proquin XR (ciprofloxacin)  [DE only] Ciloxan, Cipro, Ciprobay, Ciprobeta, Ciprodura, Ciproflox, Ciprohexal, Ehlixacin, Floxager, Keciflox (ciprofloxacin) | 🞎 | 🞎 | 🞎 |
| 6. | [US only] Floxin (ofloxacin)  [DE Only] Oculox (ofloxacin) | 🞎 | 🞎 | 🞎 |
| 7. | [US Only] Levaquin (levofloxacin)  [DE Only] Tavanic (levofloxacin) | 🞎 | 🞎 | 🞎 |
| 8. | [US Only] Augmentin (amoxicillin-clavulanate)  [DE Only] Amoxi, Amoxibeta, Amoclav (amoxicillin-clavulanate) | 🞎 | 🞎 | 🞎 |
| 9. | [US only] Omnicef (cefdinir) | 🞎 | 🞎 | 🞎 |
| 10. | Ceclor, Ceclor CD, Raniclor (cefaclor) | 🞎 | 🞎 | 🞎 |
| 11. | Banan, Vantin (cefpodoxime-proxetil) | 🞎 | 🞎 | 🞎 |
| 12. | [US only] Keflex (Cephalexin)  [DE only] Cephalex-CT (Cephalexin) | 🞎 | 🞎 | 🞎 |
| 13. | [DE only] Pivmecillinam (Selexid) | 🞎 | 🞎 | 🞎 |
| 14. | Other | 🞎 | 🞎 | 🞎 |

CQ5. In 2019, the FDA issued a drug safety announcement advising that fluoroquinolone antibiotics can increase the risk of ruptures or tears in the aorta. Please estimate your frequency of prescribing fluoroquinolone before this announcement and the frequency you now prescribe fluoroquinolone: <ASK ALL> <USA ONLY>

| 1 | Prescribing frequency before______% |
| --- | --- |
| 2 | Prescribing frequency after_______-% |

CQ5. In 2018, the Committee for Medical Products for Human Use (CHMP) confirmed the use of the remaining fluroquinolone antibiotics should be restricted with prescribing information describing the disabling and potential permanent side effects. Please estimate your frequency of prescribing fluoroquinolone before this announcement and the frequency you now prescribe Fluoroquinolone: <ASK ALL> <GERMANY ONLY>

| 1 | Prescribing frequency before______% |
| --- | --- |
| 2 | Prescribing frequency after_______-% |

CQ6. Do you feel well informed or knowledgeable about antibiotic resistance? <ASK ALL>

Please rate the statement below, where 1= not at all and to 7 a great deal.

| 1 | 2 | 3 | 4 | 5 | 6 | 7 |
| --- | --- | --- | --- | --- | --- | --- |
| ⭘ | ⭘ | ⭘ | ⭘ | ⭘ | ⭘ | ⭘ |

CQ7. Do you typically discuss antibiotic resistance with your uUTI patients? <ASK ALL>

| 1 | ⭘ | Yes |
| --- | --- | --- |
| 2 | ⭘ | No |

CQ8. How important is antimicrobial stewardship to you when treating uUTI? <ASK ALL>

**Definition:** Antimicrobial stewardship: the optimal selection, dosage, and duration of antimicrobial treatment that results in the best clinical outcome for the treatment, with minimal toxicity to the patient and minimal impact on subsequent resistance and impact on microbiome

Please rate the statement below, where 1=not at all and 7 =a great deal.

| 1 | 2 | 3 | 4 | 5 | 6 | 7 |
| --- | --- | --- | --- | --- | --- | --- |
| ⭘ | ⭘ | ⭘ | ⭘ | ⭘ | ⭘ | ⭘ |

CQ9. Please indicate to what extent you agree or disagree with the following statements in relation to antibiotic resistance in uUTI? <ASK ALL>

**Please rate the statement below, where 1=strongly disagree and 7= strongly agree.**

|  |  | 1 | 2 | 3 | 4 | 5 | 6 | 7 |
| --- | --- | --- | --- | --- | --- | --- | --- | --- |
| 1 | Antibiotic resistance is a problem elsewhere (i.e. other regions, cities, countries) and has no impact on my practice. | ⭘ | ⭘ | ⭘ | ⭘ | ⭘ | ⭘ | ⭘ |
| 2 | There is no problem, I have not seen resistance in the community setting. | ⭘ | ⭘ | ⭘ | ⭘ | ⭘ | ⭘ | ⭘ |
| 3 | Antibiotic resistance is a problem in the community setting. | ⭘ | ⭘ | ⭘ | ⭘ | ⭘ | ⭘ | ⭘ |
| 4 | There is no problem, I have not seen resistance in the hospital setting. | ⭘ | ⭘ | ⭘ | ⭘ | ⭘ | ⭘ | ⭘ |
| 5 | Antibiotic resistance is a problem in the hospital setting. | ⭘ | ⭘ | ⭘ | ⭘ | ⭘ | ⭘ | ⭘ |
| 6 | The development of antibiotic resistance is serious, and we must be careful. | ⭘ | ⭘ | ⭘ | ⭘ | ⭘ | ⭘ | ⭘ |
| 7 | I can influence the reduction in the spread of antibiotic resistance. | ⭘ | ⭘ | ⭘ | ⭘ | ⭘ | ⭘ | ⭘ |
| 8 | Antibiotic resistance influences my daily prescribing practices. | ⭘ | ⭘ | ⭘ | ⭘ | ⭘ | ⭘ | ⭘ |
| 9 | A history of antibiotic resistance in a patient influences and or changes my prescribing practices (A history of antibiotic resistant UTI infections in the same patient) | ⭘ | ⭘ | ⭘ | ⭘ | ⭘ | ⭘ | ⭘ |

CQ10. Do you have access to data on antibiotic resistance to guide empiric prescribing at the following levels: <ASK ALL>

| 1 | Yes – Local information | 🞏 |
| --- | --- | --- |
| 2 | Yes – Regional information | 🞏 |
| 3 | Yes – National information | 🞏 |
| 4 | None of the above | 🞏 |
| 5 | Don’t know | ⭘ |

CQ11. Would more readily available information on antibiotic resistance at a local, regional and/or national level be beneficial <ASK ALL>

1. For you as a physician

|  |  | Yes | No | Don’t know |
| --- | --- | --- | --- | --- |
| 1 | Local | ⭘ | ⭘ | ⭘ |
| 2 | Regional | ⭘ | ⭘ | ⭘ |
| 3 | National | ⭘ | ⭘ | ⭘ |

1. For your patients/To give to your patients

|  |  | Yes | No | Don’t know |
| --- | --- | --- | --- | --- |
| 1 | Local | ⭘ | ⭘ | ⭘ |
| 2 | Regional | ⭘ | ⭘ | ⭘ |
| 3 | National | ⭘ | ⭘ | ⭘ |

CQ12. How has COVID-19 impacted your patient management for uUTIs?

**Select all that apply**

| 1 | 🞏 | Fewer visits for individual patients (reduced visiting schedule) |
| --- | --- | --- |
| 2 | 🞏 | Only seeing more severe patients, i.e. cancelling routine appointments with mild patients |
| 3 | 🞏 | Moving to video/telephone consultation |
| 4 | 🞏 | Changed the way I choose and prescribe medication |
| 5 | 🞏 | Fewer tests/investigations performed |
| 6 | 🞏 | Limiting physical contact during consultations (e.g. blood tests or physical examinations) |
| 7 | ⭘ | COVID-19 has not impacted patient management |
| 8 | 🞏 | Other |

CQ13. How have you changed the way you prescribe medication for uUTIs?

**Select all that apply**

| 1 | 🞏 | Ensure all new prescriptions for uUTI medications are self-administered |
| --- | --- | --- |
| 2 | 🞏 | Provided prescriptions to allow for self-initiated treatment in patients with previous uUTI |
| 3 | 🞏 | Changed IV antibiotics to oral antibiotics in patients currently receiving IV treatment for uUTI |
| 4 | 🞏 | Reduced laboratory testing for uUTI |
| 5 | 🞏 | Increased frequency of empirical treatment for initial uUTI |
| 6 | 🞏 | Prescribed empirically in later lines of uUTI treatment (after treatment failure) |
| 7 | 🞏 | Prescribed a longer course of treatment |
| 8 | 🞏 | Prescribed a shorter course of treatment |
| 9 | 🞏 | Prescribed broader spectrum antibiotics as C&S testing not possible with remote consultations in those I would usually do a C&S for |
| 10 | 🞏 | Referred patient to urgent care in cases of treatment failure |

CQ14. Following the end of restrictions/social distancing do you think

| 1 |  | ⭘ | Changes in your practice will continue in case of further outbreaks |
| --- | --- | --- | --- |
| 2 |  | ⭘ | You will revert to your previous management patterns |
| 3 |  | ⭘ | You will provide a mixed approach |
| 4 |  | ⭘ | Other (specify) ____ |

SECTION D: TREATMENT SATISFACTION AND GOALS

This section addresses the level of unmet need with current treatment options to treat uUTIs considering the physicians satisfaction with current treatment options.

DQ1. Please rate your level of agreement with current oral treatment options available for uUTIs?

Please providing a rate below, where 1 = extremely disagree and 7= extremely agree

|  |  | 1 | 2 | 3 | 4 | 5 | 6 | 7 |
| --- | --- | --- | --- | --- | --- | --- | --- | --- |
| 1 | I am satisfied with the current treatment options | ⭘ | ⭘ | ⭘ | ⭘ | ⭘ | ⭘ | ⭘ |
| 2 | Knowing what treatment to select for my uUTI patients is easy | ⭘ | ⭘ | ⭘ | ⭘ | ⭘ | ⭘ | ⭘ |
| 3 | I feel there is a good selection of treatment options | ⭘ | ⭘ | ⭘ | ⭘ | ⭘ | ⭘ | ⭘ |
| 4 | I feel there is a very small selection of treatment options | ⭘ | ⭘ | ⭘ | ⭘ | ⭘ | ⭘ | ⭘ |
| 5 | My uUTI patients are knowledgeable about new treatment options | ⭘ | ⭘ | ⭘ | ⭘ | ⭘ | ⭘ | ⭘ |
| 6 | Larger diversity in treatment options would be beneficial | ⭘ | ⭘ | ⭘ | ⭘ | ⭘ | ⭘ | ⭘ |
| 7 | I am dissatisfied with the current treatment options | ⭘ | ⭘ | ⭘ | ⭘ | ⭘ | ⭘ | ⭘ |

DQ2. Please rate your level of satisfaction with current oral treatment options for uUTIs according to the following statements: <ASK ALL>

Please rate your satisfaction with current treatment options according to the following statements, where 1=extremely dissatisfied and 7= extremely satisfied

|  |  | 1 | 2 | 3 | 4 | 5 | 6 | 7 |
| --- | --- | --- | --- | --- | --- | --- | --- | --- |
| 1 | Availability of treatment options (in terms of mechanism of action) | ⭘ | ⭘ | ⭘ | ⭘ | ⭘ | ⭘ | ⭘ |
| 2 | Ability to reach the treatment goals I set for my patients | ⭘ | ⭘ | ⭘ | ⭘ | ⭘ | ⭘ | ⭘ |
| 3 | Ability to eradicate uUTI infection (microbiological cure) | ⭘ | ⭘ | ⭘ | ⭘ | ⭘ | ⭘ | ⭘ |
| 4 | Ability to provide relief of uUTI symptoms (clinical cure) | ⭘ | ⭘ | ⭘ | ⭘ | ⭘ | ⭘ | ⭘ |
| 5 | Duration of treatment | ⭘ | ⭘ | ⭘ | ⭘ | ⭘ | ⭘ | ⭘ |
| 6 | Ability of treatment options to improve quality of life | ⭘ | ⭘ | ⭘ | ⭘ | ⭘ | ⭘ | ⭘ |
| 7 | Low side effects associated with treatment | ⭘ | ⭘ | ⭘ | ⭘ | ⭘ | ⭘ | ⭘ |
| 8 | Safety concerns associated with current treatment options | ⭘ | ⭘ | ⭘ | ⭘ | ⭘ | ⭘ | ⭘ |
| 9 | Tolerability of current treatment options | ⭘ | ⭘ | ⭘ | ⭘ | ⭘ | ⭘ | ⭘ |
| 10 | Cost of current treatment options | ⭘ | ⭘ | ⭘ | ⭘ | ⭘ | ⭘ | ⭘ |
| 11 | Access to/Availability of medication | ⭘ | ⭘ | ⭘ | ⭘ | ⭘ | ⭘ | ⭘ |
| 12 | Durability of treatment options | ⭘ | ⭘ | ⭘ | ⭘ | ⭘ | ⭘ | ⭘ |
| 13 | Fast onset of symptom resolution from treatments | ⭘ | ⭘ | ⭘ | ⭘ | ⭘ | ⭘ | ⭘ |
| 14 | Treatment for patients with allergies |  |  |  |  |  |  |  |

DQ3. What are the 3 most important treatment goals for you when managing your uUTI patients? <ASK ALL>

| 1 | 🞏 | Relief of patient symptoms (Clinical cure) |
| --- | --- | --- |
| 2 | 🞏 | Clearing of infection (microbiological cure) |
| 3 | 🞏 | Education of preventing infection |
| 4 | 🞏 | Carrying out antibiotic stewardship |
| 5 | 🞏 | Prevent recurrence |

SECTION E: Barriers and drivers To treatment use

This section focuses on the barriers and drivers which influence the physician’s choice of medication and treatment including the administrative restraints.

EQ1a. Listed below are a number of attributes associated with the management of uUTI: <ASK ALL>

Please indicate how important each attribute is in terms of managing uUTI, **using a scale of 1 to 7 where 1 = of no importance and 7 = extremely important. *Enter a value for each attribute*.**

|  |  | a) |
| --- | --- | --- |
| 1 | Availability of treatment options (in terms of mechanism of action/spectrum of pathogens) | __ |
| 2 | Duration of Treatment | __ |
| 3 | Dosage of Treatment | __ |
| 4 | Ability to clear the infection (microbiological cure) | __ |
| 5 | Ability to provide relief of uUTI symptoms (clinical cure) | __ |
| 6 | Ability of treatment options to improve patient quality of life | __ |
| 7 | Patient allergies to antibiotics | __ |
| 8 | Patient adherence | __ |
| 9 | Side effects associated with treatment | __ |
| 10 | Safety concerns associated with treatment options (e.g. Black Box warnings) | __ |
| 11 | Tolerability of treatment options | __ |
| 12 | Cost of treatment to patient | __ |
| 13 | Cost of treatment to insurance | __ |
| 14 | Reducing probability of recurrent infection | __ |
| 15 | Fast onset of treatments | __ |
| 16 | Familiarity/Experience | __ |
| 17 | Patient failure on prior antibiotics | __ |
| 18 | Pathogen resistance to antibiotics | __ |
| 19 | Confirmed lab testing for susceptibility | __ |
| 20 | Local/Regional Formulary requirements | __ |
| 21 | Local/Regional antibiogram requirements | __ |
| 22 | Antimicrobial stewardship | __ |

EQ1bi) Of the attributes listed below related to the clinical management of uUTI, please rate how you believe the products/classes of products perform for uUTI in relation to each attribute, **using a scale of 1 to 7 where 1 = performs extremely poorly and 7 = performs extremely well. *Enter a value for each product for each attribute or a dash (-) if the class of product is unknown***

|  |  | Trimethoprim-Sulfamethozazole | Fosfomycin | Nitrofurantoin | [DE only] Pivmecillinam (Selexid) | Penicillins (Penams) | Cephalosporins | Fluroquinolones |
| --- | --- | --- | --- | --- | --- | --- | --- | --- |
| 1 | Availability of treatment options (in terms of mechanism of action/spectrum of pathogens) | __ | __ | __ | __ | __ | __ | __ |
| 2 | Duration of Treatment | __ | __ | __ | __ | __ | __ | __ |
| 3 | Dosage of Treatment | __ | __ | __ | __ | __ | __ | __ |
| 4 | Ability to clear the infection (microbiological cure) | __ | __ | __ | __ | __ | __ | __ |
| 5 | Ability to provide relief of uUTI symptoms (clinical cure) | __ | __ | __ | __ | __ | __ | __ |
| 6 | Ability of treatment options to improve patient quality of life | __ | __ | __ | __ | __ | __ | __ |
| 7 | Patient allergies to antibiotics | __ | __ | __ | __ | __ | __ | __ |
|  |  | Trimethoprim-Sulfamethozazole | Fosfomycin | Nitrofurantoin | [DE only] Pivmecillinam (Selexid) | Penicillins (Penams) | Cephalosporins | Fluroquinolones |
| 8 | Side effects associated with treatment | __ | __ | __ | __ | __ | __ | __ |
| 9 | Safety concerns associated with treatment options (e.g. Black Box warnings) | __ | __ | __ | __ | __ | __ | __ |
| 10 | Tolerability of treatment options | __ | __ | __ | __ | __ | __ | __ |
| 11 | Reducing probability of recurrent infection | __ | __ | __ | __ | __ | __ | __ |
| 12 | Fast onset of treatments | __ | __ | __ | __ | __ | __ | __ |
| 13 | Patient failure on prior antibiotics | __ | __ | __ | __ | __ | __ | __ |
| 14 | Pathogen resistance to antibiotics | __ | __ | __ | __ | __ | __ | __ |
| 15 | Confirmed lab testing for susceptibility | __ | __ | __ | __ | __ | __ | __ |

EQ1bii) Of the attributes listed below related to the non-clinical management of uUTI, please rate how you believe the products/classes of products perform for uUTI in relation to each attribute, **using a scale of 1 to 7 where 1 = performs extremely poorly and 7 = performs extremely well. *Enter a value for each product for each attribute or a dash (-) if the class of product is unknown***

|  |  |  |  |  |  |  |  |  |
| --- | --- | --- | --- | --- | --- | --- | --- | --- |
|  |  | Trimethoprim-Sulfamethozazole | Fosfomycin | Nitrofurantoin | [DE only] Pivmecillinam (Selexid) | Penicillins (Penams) | Cephalosporins | Fluroquinolones |
| 1 | Patient adherence | __ | __ | __ | __ | __ | __ | __ |
| 2 | Cost of treatment to patient | __ | __ | __ | __ | __ | __ | __ |
| 3 | Cost of treatment to insurance | __ | __ | __ | __ | __ | __ | __ |
| 4 | Familiarity/Experience | __ | __ | __ | __ | __ | __ | __ |
| 5 | Local/Regional Formulary requirements | __ | __ | __ | __ | __ | __ | __ |
| 6 | Local/Regional antibiogram requirements | __ | __ | __ | __ | __ | __ | __ |
| 7 | Antimicrobial stewardship | __ | __ | __ | __ | __ | __ | __ |

EQ1c) Of the products listed below, please provide an overall rating for each product/class regarding its use in managing uUTI, **using a scale of 1 to 7 where; 1 = very poor and 7 = exceptional *or a dash (-) if the class of product is unknown*.**

|  |  |  |  |  |  |  |  |  |
| --- | --- | --- | --- | --- | --- | --- | --- | --- |
|  |  | Trimethoprim-Sulfamethozazole | Fosfomycin | Nitrofurantoin | [DE only] Pivmecillinam (Selexid) | Penicillins (Penams) | Cephalosporins | Fluroquinolones |
| 1 | Overall rating (1=very poor and 7=exceptional) | __ | __ | __ | __ | __ | __ | __ |

EQ2. Which of the following guidelines do you typically use to inform your treatment decisions? <ASK ALL>

**Select all that apply**

| 1 | 🞏 | Local guidelines |
| --- | --- | --- |
| 2 | 🞏 | Regional guidelines |
| 3 | 🞏 | National guidelines |
| 4 | 🞏 | International guidelines |
| 5 | 🞏 | Peer guidance |
| 6 | 🞏 | Other (specify) ____ |

SECTION F: DEMOGRAPHICS

This section involves questions around the physicians’ setting and caseload.

FQ1. Please estimate the proportion of your time in clinic spent seeing patients with uUTIs <ASK ALL> <TOTAL CANNOT EXCEED 100%>

| _________ % of time |
| --- |

FQ2. In what type of care setting do you spend most of your patient care time? <ASK ALL>

| 1 | ⭘ | Regional Centre |
| --- | --- | --- |
| 2 | ⭘ | University/Teaching hospital |
| 3 | ⭘ | Regional/Community hospital |
| 4 | ⭘ | Private hospital |
| 5 | ⭘ | Office based practice (Public) (DE only) |
| 6 | ⭘ | Office based practice (Private) (DE only) |
| 7 | ⭘ | Private practice (US Only) |
| 8 | ⭘ | Health Centre |
| 9 | ⭘ | Urgent care |

FQ3. Which regions do you practice in? <GERMANY ONLY>

|  |  |  |
| --- | --- | --- |
| 1 | Bavaria | 🞎 |
| 2 | Lower Saxony | 🞎 |
| 3 | Baden-Wurttemberg | 🞎 |
| 4 | North Rhine-Westphalia | 🞎 |
| 5 | Brandenburg | 🞎 |
| 6 | Mecklenburg-Vorpommern | 🞎 |
| 7 | Hesse | 🞎 |
| 8 | Saxony-Anhalt | 🞎 |
| 9 | Rhineland-Palatinate | 🞎 |
| 10 | Saxony | 🞎 |
| 11 | Thuringia | 🞎 |
| 12 | Schleswig-Holstein | 🞎 |
| 13 | Saarland | 🞎 |
| 14 | Berlin | 🞎 |
| 15 | Hamburg | 🞎 |
| 16 | Bremen | 🞎 |

FQ3. In which state is your primary practice based? <USA ONLY>

| 1 | ⭘ | Show 46 other states |
| --- | --- | --- |
